# Towards the human nasal microbiome: Simulating *D. pigrum* and *S. aureus*

**DOI:** 10.3389/fcimb.2022.925215

**Published:** 2022-10-11

**Authors:** Reihaneh Mostolizadeh, Manuel Glöckler, Andreas Dräger

**Affiliations:** ^1^ Computational Systems Biology of Infections and Antimicrobial-Resistant Pathogens, Institute for Bioinformatics and Medical Informatics (IBMI), University of Tübingen, Tübingen, Germany; ^2^ Department of Computer Science, University of Tübingen, Tübingen, Germany; ^3^ German Center for Infection Research (DZIF), Partner site, Tübingen, Germany; ^4^ Cluster of Excellence ‘Controlling Microbes to Fight Infections’, University of Tübingen, Tübingen, Germany

**Keywords:** microbial communities, *Staphylococcus aureus*, *Dolosigranulum pigrum*, nasal microbiome, computational biology, genome-scale metabolic model

## Abstract

The human nose harbors various microbes that decisively influence the wellbeing and health of their host. Among the most threatening pathogens in this habitat is *Staphylococcus aureus*. Multiple epidemiological studies identify *Dolosigranulum pigrum* as a likely beneficial bacterium based on its positive association with health, including negative associations with *S. aureus*. Carefully curated GEMs are available for both bacterial species that reliably simulate their growth behavior in isolation. To unravel the mutual effects among bacteria, building community models for simulating co-culture growth is necessary. However, modeling microbial communities remains challenging. This article illustrates how applying the *NCMW* fosters our understanding of two microbes’ joint growth conditions in the nasal habitat and their intricate interplay from a metabolic modeling perspective. The resulting community model combines the latest available curated GEMs of *D. pigrum* and *S. aureus*. This uses case illustrates how to incorporate genuine GEM of participating microorganisms and creates a basic community model mimicking the human nasal environment. Our analysis supports the role of negative microbe–microbe interactions involving *D. pigrum* examined experimentally in the lab. By this, we identify and characterize metabolic exchange factors involved in a specific interaction between *D. pigrum* and *S. aureus* as an *in silico* candidate factor for a deep insight into the associated species. This method may serve as a blueprint for developing more complex microbial interaction models. Its direct application suggests new ways to prevent disease-causing infections by inhibiting the growth of pathogens such as *S. aureus* through microbe–microbe interactions.

## 1 Introduction

The human nose is home to numerous microbial species and several complex microbial ecosystems, which play a fundamental role in the wellbeing of their host (Levy and Borenstein, 2013). In fact, in nature, most microbes do not live in isolation but rather exist as part of a complex, dynamically changing microbial consortia. They continuously modify their surroundings to the benefit or disadvantage of the other organisms that live therein, thus shaping community composition and structure but also influencing the onset or progression of diseases (Clemente et al., 2012).

Microbial interactions in the human nose, as in any other environment, are complex, flexible, and capable of adapting to physiological changes. For example, changes in nutrient availability may shift the relative abundances of the community members and affect their functional capacity. Besides competition for resources, members within the community can also cross-feed each other by releasing “waste” products that are metabolized by other species. Competition for space is also likely as well as direct killing, e.g., through secretion of antimicrobial compounds (Fredrickson, 2015; Widder et al., 2016; Friedman and Gore, 2017). Cooperation and competition generate positive and negative feedback in microbial communities and influence the overall functional activities (Leung and Poulin, 2008).

Until now, over 150 different bacterial species have been identified within the nasal fluids of human subjects (Kaspar et al., 2016). The bacterial opportunistic pathogen *Staphylococcus aureus* is among the most frequently present species: in fact, it colonizes the nostrils of about ⅓ of the human population (Brégeon et al., 2018). Asymptomatic nasal carriage of *S. aureus* is a primary risk factor for developing an infection with the endogenous *S. aureus* strain, especially after surgery (Perl et al., 2002; Bode et al., 2010; Brégeon et al., 2018). The emergence and spread of MRSA pose a severe problem in treating these antibiotic-resistant infections, thus causing nearly 10,000 deaths annually in the United States alone (Centers for Disease Control and Prevention, 2008; Ramsey et al., 2016). This underlines the urgent need for research on novel antimicrobial (Conlon et al., 2013) and antivirulence therapies (Månsson et al., 2014; Murray et al., 2014; Sully et al., 2014). The factors that turn commensal *S. aureus* into a pathogen have yet to be defined. Within the nasal microbiome, the presence of *S. aureus* has been positively or negatively correlated with that of other nasal commensal species, including members of the *S. aureus* genera *Corynebacterium*, *Propionibacterium*, and *Dolosigranulum* (Uehara et al., 2000; Lina et al., 2003; Frank et al., 2010; Gardner et al., 2013; Yan et al., 2013). priority is followed by *Dolosigranulum pigrum*. has emerged in multiple studies of the human upper respiratory tract (URT) microbiota, colonizing with or without *Corynebacterium* species, as potentially beneficial and/or protective against colonization by (Laufer et al., 2011; Yan et al., 2013; Liu et al., 2015; Escapa et al., 2018; Man et al., 2020). In particular, *D. pigrum* seems to rarely co-exist with *S. aureus* in nasal microbiomes, which might prevent the colonization of the nasal cavity by *S. aureus* (Laufer et al., 2011; Yan et al., 2013; Liu et al., 2015; Escapa et al., 2018; Man et al., 2020). However, the molecular mechanisms behind these protective effects have remained largely unclear. Understanding the pairwise interactions, such as those between *D. pigrum* and *S. aureus*, as influential drivers of multi-species community dynamics is vital to manipulating microbiomes for therapeutic and prophylactic purposes (Mostolizadeh et al., 2019). However, this is often hindered by the impossibility of culturing and co-culturing the various species of the human nasal microbiota *in vitro*. Although a unique SNM3 has been developed (Krismer et al., 2014) to mimic the human nose environment, several members of the nasal microbiome remain unculturable even in this medium.

The recent advances in systems biology and *in silico* metabolic modeling have allowed the study of complex microbial communities such as the gut, skin, vagina, and respiratory tract (Chowdhury and Fong, 2020). Various modeling methodologies, such as GEM (Klitgord and Segrè, 2010; Freilich et al., 2011; Zomorrodi and Maranas, 2012; Seif et al., 2019; Diener et al., 2020), AGORA (Levy and Borenstein, 2013; Magnúsdóttir et al., 2017), CASINO (Shoaie et al., 2015), BacArena (Bauer et al., 2017), and GutLogo (Lin et al., 2018), as well as databases like the VMH (Noronha et al., 2019), have been developed to gain a quantitative understanding of how interspecies interactions may form and shape microbial communities. Concerning the nasal microbiomes, most of the studies have used amplicon-based sequencing to analyze the composition of the microbial communities in nasal swabs from healthy individuals and estimate co-occurrence relationships between the different species. In addition, *in vitro* assays have further suggested potential antagonistic or cooperative interactions among some of the members of the nasal microbiome. Furthermore, GEMs have only been constructed for a minority of bacteria of the nasal microbiome (Kaspar et al., 2016).

Unlike the human gut, the diversified landscape of the human nose provides both aerobic and anaerobic living conditions for many still uncharacterized bacteria (Proctor and Relman, 2017; Man et al., 2017). Hence, the simulation of bacterial growth within the nose requires a modification of the workflow established for the gut microbiome, which is purely anaerobic. Here, we applied our created python package named NCMW (Glöckler et al., 2022) to construct a predictive computational model of pairwise microbial community and their interactions between the published GEMs of *S. aureus* USA300 strain JE2 (Seif et al., 2019; Renz and Dräger, 2021) and *D. pigrum* strain 83VPs-KB5 (Renz et al., 2021). These GEMs are already available in SBML Level 3 Version 1 format (Bergmann et al., 2018; Keating et al., 2020; Renz et al., 2020) with flux balance constraints (FBC) extension version 2 (Olivier and Bergmann, 2018) that follows standard conventions of constraint-based models (Carey et al., 2020). *S. aureus* has been reconstructed using a genomic sequence from NCBI (Pruitt et al., 2005) *via* accession code CP020619.1, and *D. pigrum* has been reconstructed using ASM19771v1.

As metabolic exchange factors can drive morphological and developmental processes, as well as the survival of individual microbes (Phelan et al., 2012), prediction of such exchanges can provide information on whether the interacting partners promote or hinder each other’s growth, regardless of the mechanistic details of the particular species involved (Giometto et al., 2015). To our knowledge, this is the first stoichiometry-based network analysis approach applied to a human nasal microbial community. The data discussed here lay the foundation for future testing of *D. pigrum* as a potential probiotic to prevent or antagonize colonization of the nares by *S. aureus*.

## 2 Materials and methods

We begin with a brief analysis of a target organism’s genome-scale metabolic network reconstructions (GENREs). Suppose the models have been already reconstructed, manually curated, and refined. We use constraint-based modeling (CBM) to systematically compute each species’ biomass production rates in a predefined medium. A comprehensive understanding of microbial metabolism is extended from the properties of individual strains in pure culture to the combinatorial interactions supported by complex communities.

### 2.1 Quality and similarity of GEM

To check the consistency of the model for each species, we firstly use the FastCC algorithm (Vlassis et al., 2014) as implemented in COBRApy (Ebrahim et al., 2013). The next step is that the models should mimic the nutritional environment of the human nose. All species should be able to have a feasible growth in the SNM3. Each GENRE is turned into a GEM, a mathematical representation of the network. A stoichiometric matrix ***S*** is created in this process whose rows represent metabolites and columns denote reactions. Hence, FBA is implemented. The mathematical approach FBA is often used to simulate metabolisms in GENRE. FBA calculates the metabolite flow through this network. This allows one to predict the growth rate of an organism or the production rate of a metabolite, which is biotechnologically important (Orth et al., 2010). In addition, FVA is used as a computational tool to evaluate each reaction flux’s minimum and maximum range while maintaining a predefined state of the metabolic network (Gudmundsson and Thiele, 2010). With FVA, we can maintain different states, and support 90%, 70%, 30%, and 10% of the maximal possible biomass production rate to calculate the maximum and minimum for each reaction of interest.

We use the Jaccard index (also known as the Jaccard similarity coefficient) to quantify the similarity between models in terms of specific sets, the set of reactions. The Jaccard index between sets *A* and *B* is defined as (Jaccard, 1912; Tanimoto, 1958)


(1) $$J\left(A,B\right)=\frac{\left|A\cap B\right|}{\left|A\cup B\right|},$$

where *A* and *B* denote two different sets. Note that 0 ≤ *J*(*A*,*B*) ≤ 1. If *J*(*A*,*B*) = 1, then both different sets have 100% overlap; both sets are equivalent.

We assume the multi-species community as a pairwise relation to generate a multi-species metabolic model. Multi-species communities can be created in a variety of ways. Generally, these can be divided into compartmentalized, pooled, and nested approaches, each with advantages and disadvantages (Taffs et al., 2009). Here, we focus on the compartmentalized approach and briefly mention some results from a pooled approach.

### 2.2 Community structure

A *compartmentalized model* is constructed between two species using five compartments: two compartments belong to each species, and a fifth compartment represents a shared environment in which metabolites are preferentially and directly transferred to and between species (Stolyar et al., 2007; Klitgord and Segrè, 2010; Freilich et al., 2011).

For each model, a stoichiometric matrix is available. These matrices are combined with creating a new compartment (shared compartment) that communicates with each species and serves as an interface for describing environmental nutrient availability (Stolyar et al., 2007; Klitgord and Segrè, 2010; Freilich et al., 2011). For example, the model for two species would be as follows:


(2) $$\left[C_{1}\right]=\text{cytoplasm}\;\text{for}\;\text{species}\;1$$

(3) [EX1]=extracellular space of the species 1

(4) [C2] =cytoplasm for species 2

(5) [EX2]=extracellular space of the species 2

(6) [ENV]=environment shared by species 1 and 2.

We focus on the human nose as an environment. If a metabolite *X_i_* is part of this environment (*Y* and *Z* are metabolites), we add the following reactions:


(7) $$v_{i}^{EX}=\text{Exchange}\;\text{flux}\;\text{for }X_{i}:X_{i}\left[ENV\right]⇋\emptyset$$

(8) $$v_{i}^{S}=\text{Shuttle}\;\text{reaction}\;\text{for}\;X_{i}:X_{i}\left[ENV\right]⇋X_{i}\left[EX_{1}\right]$$

(9) $$v_{i}^{T}=\text{Transport}\;\text{for}\;X_{i}:X_{i}\left[EX_{1}\right]+Y⇋X_{i}\left[C_{1}\right]+Z$$

The advantage of using shuttle reactions is that they allow the monitoring of which metabolites are transported within the environment by the community of species and which ones are transported through the membrane of each species (Klitgord and Segrè, 2010). These are, however, not necessary. A compartmentalized model can also be implemented without shuttle reactions as an “integrated approach.” Nevertheless, this makes a more detailed analysis of the community impossible. Herein, we study both compartmentalized approaches, with and without shuttle reactions, differentiating the integrated model from the custom model.

**Figure 1:**
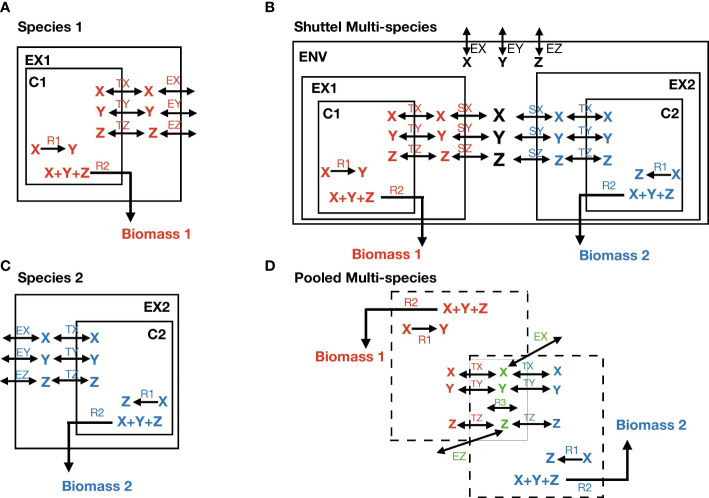
The definition of the multi-species model. **(A, B)** Show the individual model of each species. X, Y, and Z are metabolites; EX, EY, and EZ stand for exchange reactions that are present in both species; TX, TY, and TZ stand for transport reactions; R1 is a species-specific internal reaction; R2 is biomass reaction. C1 and C2 stand for cytosolic space; EX1 and EX2 for extracellular space. **(C)** Represents the construction of the shuttle multi-species model. SX stands for shuttle reactions and ENV for environmental space. **(D)** Represents the construction of the pooled multi-species model. The species-specific reactions R1 and R2, and TX, TY, and TZ appear in their own color match with the species, whereas X, Y, Z, EX, EY (not present due to lack of space), and EZ are common reactions and metabolites. They take place only once in the model with different color as green. R3 is also a common internal reaction.

Moreover, *a pooled approach* is defined as a single entity in **Figure 1D**. All metabolic reactions and metabolites from the two or three species are combined into a single compartment. Reactions catalyzed by more than one species are only considered once. All metabolic constraints of each species on SNM3 follow the original definition since there is no need for detailed knowledge of every species in the community.

### 2.3 Objective function

There exist several choices of objective functions. We specifically use the following to show the weak and strong points of separate formulations:

- The sum of the biomass reactions of the community members. This is a simple formulation to prove how important the concept of balanced growth of community members or their abundance is. Furthermore, it shows how it affects the results if one species grows faster or has a higher growth rate. To use this formula, one should carefully consider the unit definition. MBR for individual species is commonly described in units of mmol/(gDW[species]·h). In contrast, the growth rate of the entire microbial community is formulated in units of mmol/(gDW[total]·h). Hence, we herein computed the biomass of each species in SNM3 by adding constraints such as uptake rate of nutrients defined in SNM3 to 10 except oxygen and iron to 20 mmol/(gDW·h) and 0.1 mmol/(gDW·h), respectively. Therefore, the unit is defined as a yield, *V*_biomass/nutrients_, with units of grams of biomass per mole of nutrients constrained (Stolyar et al., 2007; Freilich et al., 2011; Gottstein et al., 2016). This approach shows the co-culture interaction between species.
- The sum of biomass reactions of the community members with different coefficients: This is given $V=\sum_{i=1}^{n}w_{i}\cdot V_{{\text{model}}_{i}}$ where *w*_*i*_∈*ℝ* ^+^∪{0} is the weight (or objective coefficient) for each species. The weights *w_i_* can be defined as the abundance of each species (Gennert and Yuille, 1988; Freilich et al., 2011). This approach is suitable to be experimentally used if the relative abundances of bacterial taxa in human nose are available.
- While keeping the total biomass constant equal to 0.1 (1/h), the objective function is α% of total biomass in the first species and (1-α)% of total biomass in the second species (El-Semman et al., 2014). The biomass reaction *V_BM_* is usually normalized such that it will produce 1 g of biomass, which results in unit 1/h corresponding to the organism’s growth rate. This approach simulates how a composition of two species interact and in which weight (abundance) the interaction will be changed. In addition, this can be matched with what one can experimentally observe as an abundance ratio.
- The same as the second formulation with additional constraint to enforce all growth *V*_model_*i*__ ≥ *V* ^*min*^. Hence, there will be no feasible solution if a single model cannot obtain *V^min^*. Alternatively, we ensure that each community member reaches a certain percentage of the total community growth, *w*_*i*_ *V*_model_*i*__ ≥ *α V* with *α*∈[0,1] (Diener et al., 2020). This allows us to “close” all shuttle reactions if we set *w_i_* = 0, guaranteeing the absence of influx or efflux at the corresponding model *i*. This approach can be complementary to the second scenario when a taxon is available; it is assumed to grow.

A variety formulation for the community objective could help us understand the importance of the chosen objective function. In addition, this indicates how difficult it is to define a community objective function meaningfully in both biological and mathematical terms. Furthermore, if the aim is only to observe the co-culturing of two species, the first technique can help match the experimental and computational. If the abundance data of taxa in a community are available, their abundance along the second scenario can be used to observe a more realistic community interaction. Suppose co-culturing is of interest while there is no further information about the abundance ratio in which the interaction is switched. In that case, the third scenario is helpful on how the experimental should apply a head start for the species with more abundance. The last technique is proper when we observe all available species in the community achieve specific growth. Multiple efforts have been made to classify the currently used multi-objective optimization techniques. First of all, it is essential to distinguish the two stages in which the solution of a multi-objective optimization problem can be divided: optimizing the objective functions involved and deciding what kind of trade-offs are appropriate from the decision-maker perspective (the so-called multi-criteria decision-making process). This section discusses some of the many techniques available for these two stages by analyzing some of their advantages and disadvantages. Multi-objective optimization problems come up. The set of optimal solutions (Pareto front) has to be identified using an effective and complete search procedure. This allows the decision-maker and the designer to carry out the best choice. The most popular classification of techniques proposed by Cohon and Marks (1975) are as follows (Chiandussi et al., 2012):

Priori technique: Take decisions before searching, which includes approaches that assume that the decision-maker can perform a particular desired achievable goal or a certain pre-ordering of the objectives prior to the search.

Posteriori technique: Search before making decisions that do not require prior preference information from the decision-maker.

Progressive technique: Integrate search and decision-making, which generally find a set of non-dominated solutions. This means that this set of solutions is non-dominated to each other but superior to the rest of the solutions in the search space. Thus, get the decision-maker’s reaction regarding this set of non-dominated solutions and modify the preferences of the objectives accordingly. Repeat the two previous steps until the decision-maker is satisfied or no further improvement is possible.

### 2.4 Media

The main medium applied herein is SNM3, where the lower bound to the reactions exchanging metabolites that are present in the SNM3 was set to −10 mmol/(gDW·h) except −20 mmol/(gDW·h) and −0.1 mmol/(gDW·h) for oxygen and iron, respectively. The lower bound to all other exchanges was set to 0. We assumed that the growth for each species in SNM3 could be achieved. This medium was throughout utilized as the primary medium for the community. In addition, we executed two more media as subsets of SNM3 for the multi-species community; one medium as rich in the nutrients of the human nose and the other one as poor in the nutrients. *Rich medium* has been defined as COMPM (Freilich et al., 2011) computed by the union of the exchange reactions between two species. The flux range is set to the minimum required amount, such that each species obtains the MBR alone. FVA was applied to compute it. Formally, this medium can be defined as follows (Freilich et al., 2011):


(10) $$\left\{V_{\text{COMPM},AB}\right\}=\left\{V_{\text{COMPM},A}\right\}\cup \left\{V_{\text{COMPM},B}\right\}$$

(11) $$V_{i,\min\_\text{FVA},AB}=\min\;(V_{i,\min\_\text{FVA},A},V_{i,\min\_\text{FVA},B}),$$

where *V*_COMPM,*AB*_ denotes COMPM for the multi-species community, which allows each species to reach its MBR in SNM3, and *V*_COMPM,*A*_ is COMPM for each species. In addition, *V*_*i*,*min*_FVA_ denotes the lower limit (maximal flux of metabolites into a compartment) of a given reaction in SNM3.

The second medium was named the poor medium. This has been defined as COOPM (Freilich et al., 2011), a medium with a minimal set of metabolites that allows the multi-species system to obtain only a positive growth rate, but not MBR. If any metabolite from the set is removed, the system would not have such a solution. Since this medium was introduced as a poor medium, species could only obtain 10% of MBR. This can be calculated using mixed-integer linear programming as follows (Burgard et al., 2001; Suthers et al., 2009; Freilich et al., 2011):


(12) $$\begin{array}{r} \begin{array}{c} \max\;Z=\sum_{i=V_{i,\text{COMPM},AB}}^{n}\theta_{i}\\ \text{Subject}\;\text{to}:\\ SV=0 \end{array}\\ V_{BM,\text{COOPM}}\geq \frac{V_{BM,\text{COMPM}}}{10}\\ \begin{array}{c} V_{BM,\text{COOPM,}AB}+V_{i,\min}\theta_{i}\geq V_{i,\min}\\ \theta \in \left\{0,1\right\}\\ i\;\in V_{i,\text{COMPM},AB\cdot } \end{array} \end{array}$$

The maximization problem is included, finding a set of exchange reactions through all metabolites in { *V*_*i*,COMPM,*AB*_ } with a constraint on minimal growth rate *V*_*BM*,COOPM_ ≥ *V*_*BM*,COMPM_/10, where *V*_*BM*,COOPM_ and *V*_*BM*,COMPM_ are the biomass rate on COMPM and COOPM, respectively. The constraint *V*_*i*,COOPM,*AB*_+*V*_*i*,*min*_ *θ*_*i*_ ≥ *V*_*i*,*min* _ represents whether or not an exchange reaction metabolite *i* is consumed. If metabolite *i* is consumed, *V*_*i*,COOPM,*AB*_ ≤ 0, then the binary variable *θ*_*i*_ attains 0 and vice versa.

### 2.5 The application of objective functions on different media

To compute the maximal biomass rate of the whole community, we applied each objective function defined at a time in combination with different media.

When the compartmentalization or pooled approaches expressed in **Figure 1** was used, the environment compartment was supposed to be SNM3 or either COMPM or COOPM. In addition, we applied using the method OptCom (Zomorrodi and Maranas, 2012). OptCom is a multi-level and multi-objective optimization formulation for metabolic modeling with flux balance analysis for the microbial community. OptCom considers each species’ biomass maximization problem as a separate inner problem. Moreover, it makes an outer optimization problem on the community level. The outer problem represents the exchange of metabolites among different species. OptCom was implemented only for the compartmentalized approach with the definition shown in **Figure 1C**. The inner problems are subsequently linked with the outer stage through inter-organism flow constraints and optimality criteria to optimize a community-level (e.g., overall community biomass) objective function. Overall, OptCom finds the set of growth rates that maximize community growth and individual growth simultaneously. This optimization problem is a multi-objective and multi-level optimization. There are several techniques to solve this type of optimization.

In addition, we used the package MICOM (Diener et al., 2020). MICOM is also a mathematical modeling framework similar to the formulation used in OptCom (Zomorrodi and Maranas, 2012). MICOM assumes the growth rate in steady state to always be available for each species and that the relative abundance in the community is in a steady state. Furthermore, MICOM supposes that the coefficient (distribution) of any species in the outer objective function is not the same, and it depends on the abundance of species. Therefore, this coefficient is defined as the relative abundance of the associated species. Another package named SteadyCom (Chan et al., 2017) also follows the same aim with a slight difference. This predicts microbial abundances from a list of taxa present in a sample by assuming the same growth rate of all species (Chan et al., 2017), whereas MICOM requires abundances as input. MICOM achieves the results in two steps: the first step is an optimization that maximizes only the community growth rate by using the growth rate distribution. Thus, the second step is followed by applying the regression by minimization the L2 norm of individual growth rates as the cooperative trade-off strategy with various levels of suboptimality ranging from 10% to 100% of the maximum community growth rate.

### 2.6 Interaction prediction

Finally, we can determine the interaction between two species computationally. Freilich et al. determined the level of competition and cooperation between species by comparing their individual and combined biomass rate across simulated communities on the COMPM and COOPM. They designed two different formulas as a PCMS and a PCPS, to quantify the level of competition and cooperation predicted among the species.


(13) $${\text{PCMS}}_{AB}=1-\frac{V_{BM,COMPM,AB}-max(V_{BM,COMPM,A},V_{BM,COMPM,B})}{V_{BM,COMPM,A}+V_{BM,COMPM,B}-max\;(V_{BM,COMPM,A},V_{BM,COMPM,B})}$$

(14) $${\text{PCPS}}_{AB}=1-\frac{V_{BM,COOPM,A}+V_{BM,COOPM,B}}{V_{BM,COOPM,AB}}$$

where *V*_*BM*,*x*,*y*_ represents the maximal biomass rate of species *y* in community *x*. If the PCMS value equals 0, it denotes no competition, while 1 indicates maximal competition. In addition, the negative PCMS values and positive PCPS values stand for cooperation, while negative PCPS values indicate competition.

## 3 Results

The GEM for *S. aureus* herein applied (Seif et al., 2019) is based on the genome sequence of *S. aureus* USA300 strain JE2. This MRSA strain contains 854 genes, 1,440 reactions, 1,327 metabolites, and 673 three-dimensional protein structures. The GEM for *D. pigrum* applied herein (Renz et al., 2021) is based on the *D. pigrum* strain VPs-KB5, which contains 854 genes, 1,670 reactions, and 1,239 metabolites.

### 3.1 The GEMs of *D. pigrum* and *S. aureus*

We started with GEM, which have already been reconstructed, manually curated, and refined. The models were also curated using the FastCC algorithm implemented in COBRApy (Ebrahim et al., 2013). These more consistent models obtained a total score of 87% for *D. pigrum* and 80% for *S. aureus.*

It has been reported that both bacteria grow on SNM3, a medium mimicking the environment of the human nose (Krismer et al., 2014). For the simulation, the exchange flux bounds were set to lie between −10 mmol/(gDW·h) and 1,000 mmol/(gDW·h) except for oxygen and iron, which were set to −20 mmol/(gDW·h) to 1,000 mmol/(gDW·h) and −0.1 mmol/(gDW·h) to 1,000 mmol/(gDW·h), respectively. This setting was throughout used as a default setting. The original model for *D. pigrum* had no growth on SNM3, as Renz et al., 2021 reported. Renz et al., 2021 achieved growth by identifying the missing metabolites that supplemented the medium allowed growth of the organism on SNM3. The missing metabolites include the amino acids L-isoleucine and L-methionine and 2,6-diaminoheptanedioate, which were required for peptidoglycan metabolism (Renz et al., 2021). The simultaneous addition of the three metabolites allowed *D. pigrum* to possible grow by 0.28 mmol/(gDW·h) on SNM3. In contrast, the original model of *S. aureus* (Seif et al., 2019) achieved a growth of 2.55 mmol/(gDW·h) on SNM3.

**Figure 2:**
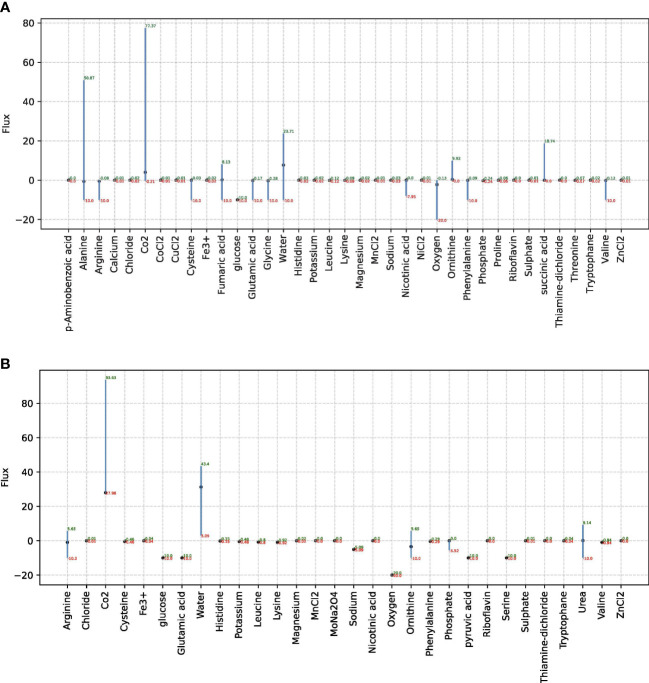
The FVA results for non-zero exchange reactions in species *D. pigrum* and *S. aureus*. In each plot, only non-zero exchange reactions that can take place in SNM3 are taken into account. FVA returns the boundaries for the fluxes through each reaction that can, paired with the right combination of other fluxes, estimate the MBR. Reactions that can support a low variability of fluxes through them are likely to be of higher importance to an organism and FVA is a promising technique for the identification of important reactions. The *x*-scale in each plot shows the associated metabolites that considered exchange reactions and the *y*-scale shows the respective flux for the related exchange reaction. **(A)** Is associated with *D. pigrum* while **(B)** Stands for the species *S. aureus.*

The FBA and FVA were implemented for each model to mimic the environment of the human nose. The associated plots for all non-zero exchange fluxes are shown in **Figure 2A** for *D. pigrum* and in **Figure 2B** for *S. aureus.*

To compute realistic growth values in the human nose for each organism, we considered the default setting on the exchange reactions and examined the growth for uptake bounds that are scaled by a multiplication factor *k*∈{ 1,…,110 } (**Supplementary File—Figure** “growth_scaled”). This helped us compare the growth rate of both species and estimated how differences in the abundance of each species could change its growth rate.

### 3.2 The uptake and secreted metabolites for *D. pigrum* and *S. aureus*

Multiple studies have experimentally shown that *D. pigrum* inhibits *S. aureus* (Brugger et al., 2020; Janek et al., 2016), but the underlying mechanisms remain unclear. Therefore, we identified metabolites that are either secreted or taken up by each species to identify possible metabolic interactions between them (see **Supplementary File**). The Jaccard index between all uptake reactions is 0.59. However, there are commonalities between both species regarding the specific metabolites they absorb (**Table 1**).

**Table 1:** Common uptake metabolites between *D. pigrum* and *S. aureus* with the relevant descriptive name extracted from the BiGG Models Database (Norsigian et al., 2020).

Common uptake metabolites			
arg__L	his__L	na1	so4
cl	k	nac	thm
cys__L	leu__L	O2	trp
fe2	lys__L	phe__L	val__L
glc__D	mg2	ribflv	zn2
glu__L	mn2		

To determine how altering the common uptake reactions affects the growth of each individual species, we set the lower bound of common uptake reactions to [minFVA, 0] and then plotted the model growth behavior against this lower bound restriction for each species (**Supplementary File—Figures** TEST_secretion_uptake_DP_flux_growth and TEST_secretion_uptake_SA_flux_growth). This resulted in, for some metabolites, a minimal uptake that was sufficient to obtain non-zero growth, while for some others, a reduction in uptake could lead to a linear or exponential drop in growth as they were in isolation. In contrast, *S. aureus* took up only one metabolite ornithine secreted by *D. pigrum*. Nonetheless, the dependency of *S. aureus*’ growth on this metabolite was low (**Supplementary File—Figure** Common_uptake_secretion_No_community), and this metabolite was already available in SNM3. Therefore, this suggested that a beneficial interaction between the two species is unlikely.

In contrast, the finding that the two species compete for uptake of common metabolites, above all those needed in high amounts to reach maximal growth (**Supplementary File—Figure** com-mon_uptake_No_community), strengthens the assumption that these two species may inhibit, as was experimentally demonstrated (Brugger et al., 2020; Janek et al., 2016). However, we cannot exclude that the behavior of each species changes in the community context, which is more physiologically relevant. We set out to address this issue with an analysis on the community level.

### 3.3 The artificial design of the pairwise community between *D. pigrum* and *S. aureus*

To further analyze the interaction between these two species, we artificially designed a pairwise community between *D. pigrum* and *S. aureus*. Hence, we started with some comparisons between both organisms. We found 377 overlapping and 970 unique metabolites between these two species. Moreover, there are 273 common and 1,535 unique reactions. Therefore, we calculated the Jaccard index to find the similarity between reactions and metabolites of both species. The Jaccard index between reactions was 0.151 and that between metabolites was 0.279. Although those values were small, they showed resource overlap between *D. pigrum* and *S. aureus*.

To compute the rich and poor media for *S. aureus* and *D. pigrum*, we first calculated the common exchange reactions between both species (**Supplementary File**, see common exchange reactions) and defined their flux as the minimum of minimal FVA fluxes of both species. Both species would not achieve growth based on the list of common exchange reactions. *S. aureus* needs four additional metabolites associated with exchange reactions EX_urea_e, EX_pyr_e, EX_mobd_e, and EX_ser__L_e. While the missing exchange reactions for *D. pigrum* are 11 exchange reactions as EX_gly_e, EX_cu2_e, EX_succ_e, EX_thr__L_e, EX_4abz_e, EX_ala__L_e, EX_ca2_e, EX_ni2_e, EX_pro__L_e, EX_fum_e, and EX_cobalt2_e.

Therefore, the common exchange reactions computed by FVA could not be a shared medium in the community for both species due to not supporting the growth of every single one. Since it is unlikely that a community with only common exchange reactions between two species supports growth, we defined a community whose medium is the union of all exchange reactions computed by FVA for both species. The defined boundary of the exchange bounds followed the default setting of bounds. This medium was called an exchange medium and included 111 exchange reactions. By this medium, a very rich and huge community was constructed. This allowed observing more details of the interaction between species but had disadvantages, such as not being feasible for experimental validation due to the enormous number of required metabolites. Additionally, since the minimum and maximum flux for some exchange reactions are the same and equal to zero, those can be neglected from the medium. Therefore, the number of metabolites in the final version of the medium is incredibly reduced.

### 3.4 Compm

To improve the exchange medium, we continued with COMPM. As already described, this medium consists of the union of exchange reactions in both species. However, the exchange flux bounds were defined in a different way to reduce the number of metabolites that support the maximum growth of each species. We thus defined the flux as a minimum of min FVA fluxes for those exchange reactions that are common and only took the corresponding min FVA flux for uncommon ones. Therefore, COMPM was computed by taking the pairwise minimum of the minimal fluxes given by FVA. The resulting medium was saved as a JSON file (**Supplementary File**—JSON file of COMPM). This medium consisted of a combination of common exchanges plus 4 required metabolites for *S. aureus* and 11 required ones for *D. pigrum*. In the community model constrained with this medium, both species achieved their maximal biomass rate in SNM3 without the other.

In the process of pairwise relationships, we also computed COOPM, a poor medium, using Equation (12). Since the calculation of this medium depended on the MBR of the community computed in COMPM, this will be explained in a few steps later. To summarize, we take into account four media in the construction of the pairwise community; SNM3 as the main medium and exchange medium, COMPM, and COOPM, whose bases are subsets of SNM3.

### 4.5 The computation of the MBR of the compartmentalized community as an integrated model

To compute the maximal biomass rate of the community with all different defined media, we constructed a community model as follows:

- We named each non-exchange reaction and associated metabolites or compartments by id + model_id, where model_id is the identifier of the model. Thus, COBRApy considered them as separate systems.
- We added the exchange metabolites and reactions unchanged as the environment should be shared by both models. We defined the set of all exchange reactions as the union of the sets from each single species.
- The community objective with adding weights to the linear combination of the growth rate of each species was implemented. Hence, this is given by $V=\sum_{i=1}^{n}w_{i}\cdot V_{model_{i}}$ , in which *w_i_* is the weight for each species (Freilich et al., 2011). The weight can be defined as the abundance of each species.

We started this approach with SNM3 as the main medium. This computation resulted in the fact that with weight 1:1, mostly *S. aureus* grows; the growth rate of *D. pigrum* is 0.024 mmol/(gDW·h), while that of *S. aureus* is 5.025 mmol/(gDW·h). This happened because the standard growth of *S. aureus* is approximately 10 times higher than that of *D. pigrum*, and assuming the same abundance for these species maintained the non-realistic formulation and results. To compensate for the slower growth rate of *D. pigrum* and justify this difference, we give a larger weight (coefficient factor) to *D. pigrum* in comparison with *S. aureus*. This shows the role of a high abundance of *D. pigrum* in the healthy upper respiratory tract (URT), which has mainly been found in several studies (Brugger et al., 2020; Laufer et al., 2011; Biesbroek et al., 2014; Lappan et al., 2018; Gan et al., 2019). Hence, we set the weight to 10:1 for *D. pigrum*: *S. aureus*. This was already examined *in vitro* in (Brugger et al., 2020) by giving a head start to *D. pigrum*. In this situation, the growth of *S. aureus* is 3.842 while *D. pigrum* grows 0.286 mmol/(gDW·h). The MBR of the community is equal to 10 MBR_*DP*_+MBR_*SA*_ = 10 (0.286)+3.482 = 6.341.

Of note, we computed the biomass of each species in SNM3 by adding constraints such as uptake rate of nutrients defined in SNM3 to 10 except oxygen and iron, as was already explained. Additionally, the fact is that FBA does not predict kinetic rates, while FBA predicts the flux distributions that provide the maximal yield on the limiting nutrient. Therefore, if the uptake rate of a nutrient, e.g., v_glucose_, is set to a particular value and objective function such as growth rate or biomass is maximized, then the optimal values of the remaining v_j_ are found. Hence, the fluxes are computed to maximize the ratio MBR/*v*_glucose_. As mentioned before, this expression is defined as a yield, *V*_biomass/glucose_, with unit of grams of biomass per mole of glucose (Gottstein et al., 2016). This implementation caused the effect of biomass abundance based on the ratio appearing in the community for both internal and exchange reactions (even shuttle exchange reactions in the next chapter). In this situation, only the ratio of weights was essential and affected the community. Therefore, if one uses the normalized scale for weights such as 0.5:0.5 instead of 1:1 or 0.909:0.091 instead of 10:1, then the flux taken up or secreted by each species in the community is the same since these weights show a ratio of 1 or 10 times more [see the **Supplementary (CSV) Files**].

We continued this examination as we changed the medium to exchange medium. With both weights, 1:1 and 10:1 for *D. pigrum*: *S. aureus*, both species achieved their maximum growth rate, i.e., *D. pigrum* equals 0.282 mmol/(gDW·h), and *S. aureus* is 2.558 mmol/(gDW·h). Of course, the community growth achieved MBR*_DP_* + MBR*_SA_* = (0.282) + 2.558 = 2.841 with weight 1:1 and 10 MBR*_DP_* + MBR*_SA_* = 10 (0.282) + 2.558 = 5.386 as the weights assumed with the ratio 10:1. In this situation, when we used the same abundance for both species, *D. pigrum* could also grow. Notably, the medium used is large with all essential and non-essential metabolites. Therefore, it is likely that each species behaved so that other species are not there due to a vibrant environment. This situation might not be possible to examine experimentally because many metabolites are required.

To calculate the community growth using the COMPM medium, we replaced the medium with COMPM in the community by an integrated model. With the weight 1:1 for *D. pigrum*: *S. aureus* reached its maximum biomass rate as 2.558 mmol/(gDW·h), while *D. pigrum* had no growth, as was expected due to the same abundance, which is not biologically meaningful. The results with COMPM using weight 10:1 for *D. pigrum: S. aureus* are 2.175 mmol/(gDW·h) for *S. aureus* and 0.072 mmol/(gDW·h) for *D. pigrum*, while the community growth is represented as 10 MBR*_DP_* + MBR*_SA_* = 10 (0.072) + 2.175 = 2.904.

**Figure 3:**
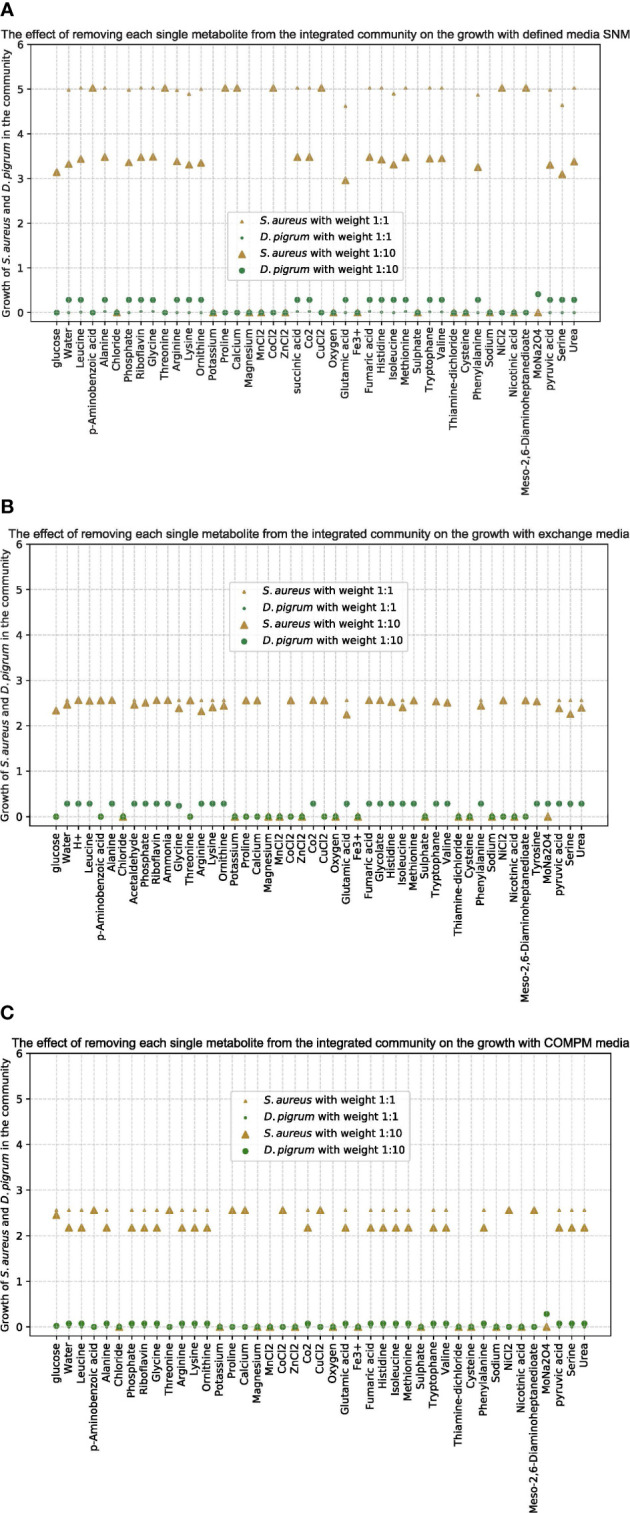
The effect of removing each single metabolite from the community by the integrated model on the growth of species *D. pigrum* and *S. aureus* as few different media implemented. **(A)** Is for the defined SNM3. **(B)** Is when the medium is defined as exchanges computed by FVA. **(C)** Is for COMPM defined. The objective function in the community is defined as a liner combination of the growth of both species. With all different media, once the weights in the objective function are assumed equally as 1:1 and one more time as 10:1 for *D. pigrum*: *S. aureus.* The small ◦ and △ are for the 1:1 ratio in the objective function and big ◦ and △ are for the 10:1 ratio. The gold color represents the growth of *S. aureus* in the community by the integrated model and the green one is for *D. pigrum*. The *x*-scale shows the metabolites in the defined medium and the *y*-scale shows the growth of each species in the community by the integrated model.

To further analyze the importance of some metabolites for the community, we removed each one and monitored how the growth of each single species in the community was affected. As shown in **Figure 3**, the growth of both species in the community was affected by the absence of some metabolites such as trace elements, independently of the applied medium.

The construction of the community as an integrated model makes further analysis more difficult. To solve this problem, we implemented a compartmentalized community in the next section.

### 3.6 The computation of maximal biomass rate of the compartmentalized community as a custom model

To construct a compartmentalized community using shuttle reactions, we used the approach presented in **Figure 1C**. We implemented a custom model with the FBA SciPy linear programming solver (Virtanen et al., 2020). The shuttle reactions were introduced using a special block matrix representation of the stoichiometry matrix. We created two environments for the shuttle reactions. The first one uses all 111 exchange reactions computed by FVA as shared shuttle reactions. The other one was restricted to all uptake and secretion reactions computed from the model summary by FBA. This diversity conveys the way to look more precisely into the community. Moreover, the objective function was again defined as the weighted sum of individual biomass functions. We weighted them such that the individual growths are balanced (i.e., *D. pigrum* gets a weight of approximately 10). With both approaches, i.e., all 111 exchange reactions or shared uptake and secretion reactions as shuttle reactions, the growth rates for every single model in the community and the community growth are computed and shown in **Table 2**. As long as *D. pigrum* had the lowest weight, regardless of the employed medium, its ability to fight for common resources remained low, thus resulting in higher growth of *S. aureus*. Therefore, *S. aureus* got the higher amount of each common uptake, showing growth, while *D. pigrum* did not. This again strengthened the assumption that using the same abundance for species contributed in the community might lead to the non-significant biological results. Therefore, one must consider that if the simple formulation for objective function is used, then the biomass abundance should be taken into account for exchange fluxes. Otherwise, this formulation cannot be correct (Gottstein et al., 2016). We skipped this implementation due to assigning different weights to *S. aureus* and *D. pigrum*. However, we tried to show it in each step to indicate not achieving meaningful results. Therefore, by assigning the weight 10 to *D. pigrum, D. pigrum* could even reach its MBR. Since one advantage of the compartmentalized approach with shuttle reaction is to define the shared environments of interest, we defined the shared environment as only oxygen being shared and observed that the growth of *S. aureus* was reduced. However, in this community, with only oxygen being shared, *D. pigrum* could grow.

**Table 2:** Comparison of growth rates of species *D. pigrum* and *S. aureus* and their community growth in the compartmentalized community.

111 total exchange reactions and all shared uptake and secretion reactions				
	Growth	SNM3	Exchange	COMPM
Weight 1:1	*S. aureus*	2.558	2.298	2.558
	*D. pigrum*	0.000	0	0.000
	Community	2.558	2.298	2.558
Weight 10:1	*S. aureus*	1.471	1.208	2.175
	*D. pigrum*	0.282	0.282	0.072
	Community	4.294	4.032	2.904

In this situation, shuttle reactions for the 111 exchange reactions and all shared uptake and secretion reactions are implemented. Weights 1:1 and 10:1 for *D. pigrum*: *S. aureus* are applied. The unit for all growth values is mmol/(gDW·h).

Further analysis on shuttle reactions is represented as the contribution of each shuttle reaction in any species when they are in the community (see the summaries of exchange in the supplement in CSV format).

To define different objective functions, we implemented an optimization for fixed community biomass rate, where *D. pigrum* and *S. aureus* attained *α*% and (1−*α*)% of the biomass, respectively, as discussed in *Section 2*. Therefore, we fixed the MBR of the whole community to 0.1 mmol/(gDW·h) to allow each species to gain *α*% and (1 − *α*)% of MBR of the whole community. This was achieved by adding two constraints for several values of α ∈ (0, 1):


(15) $$\begin{array}{c} 0.1=\max\;\sum_{m\in \left\{SA,DP\right\}}V_{BM,m}=\max\;\;\left(V_{BM,DP}+V_{BM,SA}\right)\\ Subject\;to:\\ \begin{array}{c} V_{BM,SA}\leq \alpha \cdot 0.1,\\ V_{BM,DP}\leq (1-\alpha )\cdot 0.1, \end{array}\\ \alpha \in (0,1) \end{array}$$

The results for several values of α ∈ (0, 1) in different scenarios were analyzed. In the first scenario, all 111 exchange reactions were defined as shuttle reactions with weights 1:1 and 10:1 for *D. pigrum*: *S. aureus* as we are implementing a few different media. The results are represented in the **Supplementary File** (see the summaries of exchange in CSV format).

**Figure 4:**
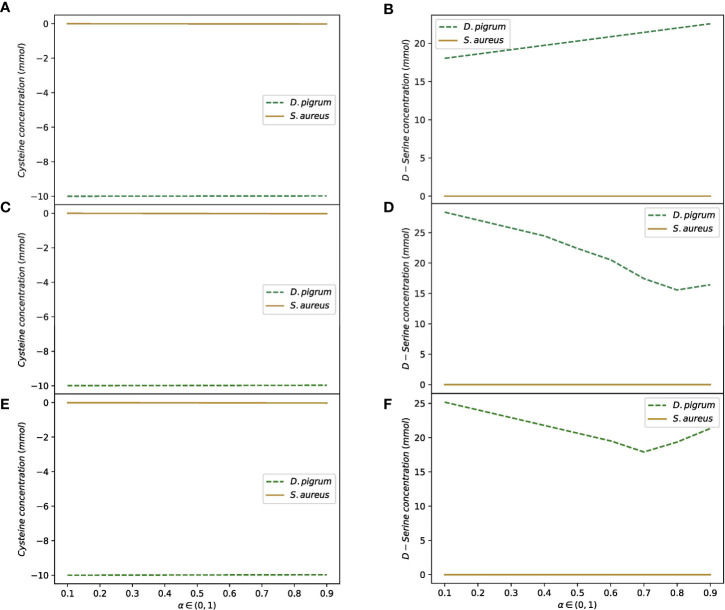
D-serine is released by *D. pigrum* as the compartmentalized community model with shuttle reactions is used. The convex objective function is implemented. The medium is defined for SNM3, exchange, and COMPM. The release of D-serine does not depend on all different media used. This is released in high amounts in all different settings of the community as α in the objective function is changed in the range of (0, 1). Cysteine is also taken up by *D. pigrum. S. aureus* does not depend on the amount of uptake of cysteine or released D-serine. **(A)** Shows how cysteine is taken up by *D. pigrum* while *S. aureus* is not influenced by cysteine while **(B)** Shows how D-serine is released by *D. pigrum* as the defined medium is SNM3. **(C, D)** Denote the uptake of cysteine by *D. pigrum* while *S. aureus* is not influenced by cysteine and secreting D-serine by *D. pigrum* as exchange medium is defined. **(E, F)** Indicate the uptake of cysteine by *D. pigrum* and no influence of cysteine, as well as the release of D-serine by *D. pigrum* while the medium is defined as COMPM.

In the second scenario, the shuttle reactions comprised all shared uptake and secretion reactions with few different media. The results for the weights 1:1 and 10:1 for *D. pigrum*: *S. aureus* are shown in the **Table 2**. All different scenarios indicated that the more weight is given to *D. pigrum*, the more *D. pigrum* grows at the expense of *S. aureus*. This showed that assigning the abundance of species in the implementation of the objective function is a significant feature that must be considered (Zomorrodi and Maranas, 2012; Chan et al., 2017; Diener et al., 2020). The contribution of each species to the consumption or release of the shared compounds in the community is displayed. **Figure 4** shows that when two species are in the community, their behavior changes in comparison to when they are analyzed in isolation. For instance, D-serine was not released by *D. pigrum* when it was in isolation, as shown in the **Supplementary File** (see the uptake and secretion of *D. pigrum* and *S. aureus*), while it was released by *D. pigrum* when it was considered in a community (see the **Supplementary CSV Files**). **Figure 4** represents how D-serine was released in all different media for the compartmentalized model with shuttle reaction.

### 3.7 The computation of maximal biomass rate of pooled community

In the previous section, we created compartments to separate both species. Herein, as was represented in **Figure 1D**, we threw all reactions into one bucket. Those reactions that are present in both species appeared once. All settings relevant to SNM3 were already implemented on both models. By using this approach with weight 1:1 for *D. pigrum*: *S. aureus*, the growth obtained for *D. pigrum* was 0.349 mmol/(gDW·h) and 5.010 mmol/(gDW·h) for *S. aureus*. This could happen due to one fully rich bucket containing all essential and non-essential metabolites (Stolyar et al., 2007; Freilich et al., 2011; Gottstein et al., 2016). With weight 10:1 for *D. pigrum*:*S. aureus*, *D. pigrum* reached 1.340 mmol/(gDW·h), *S. aureus* failed to grow [0.0 mmol/(gDW·h)], and the growth of the community equaled to 10 MBR_DP_ + MBR_SA_ = 10 (1.340) + (0.0) = 13.402. The advantage of the pooled reaction approach was the reduction of computational burden as the code was written and implemented efficiently. In addition, this method represented a flexible starting point for analyzing the community of *D. pigrum* and *S. aureus* when the well-documented experimental work is missing. This analysis supports the assumption that *D. pigrum* inhibits *S. aureus* growth, but a larger weight for *D. pigrum* is required to achieve this condition. In contrast, the pooled community analysis failed to determine whether the species employed a particular enzyme or produced biomass. Likewise, the information about how the metabolites were transferred between species was unclear.

### 3.8 The computation of maximal biomass rate by implementing the OptCom community on MICOM

In the next step, to compare the results achieved by already available tools, we used the OptCom (Zomorrodi and Maranas, 2012) community objective implemented in the MICOM (Diener et al., 2020) package. Due to the initially constructed MICOM for the models on the human gut and AGORA models, directly using MICOM is not possible. We re-wrote the MICOM with our settings, such as the definition of SNM3 used, fixing the boundaries that continuously switch to the default setting of MICOM, plus resetting the effect of abundances on the flux distribution. We observed the same results after using these settings and skipping the conflicts faced in each time run, which could be ascribed to numerical errors. For instance, MICOM with the relative abundance of 0.5 and 0.5 showed a growth of 2.71 mmol/(gDW·h) for *S. aureus* and no growth for *D. pigrum*. We considered only one metabolite, for instance, EX_cys__L_e, for examination. However, these flux distributions computed by FBA only provide the maximal yield on the limiting nutrient. *S. aureus* consumed 0.27 mmol/(gDW·h) of this metabolite, while *D. pigrum* consumed 9.39 mmol/(gDW·h). Hence, the overall exchange shuttle was (−0.27).(0.5)+(−9.39).(0.5)=−4.8 (see the **Supplementary CSV Files**).

We used the weights 0.5 and 0.5 for both species to compare them with our approach. Therefore, *D. pigrum* had no growth while *S. aureus* grew 2.55 mmol/(gDW·h). The flux value of 9.39 mmol/(gDW·h) consumed by *S. aureus* was 0.46 mmol/(gDW·h) and that by *D. pigrum* was 9.53 mmol/(gDW·h). This happened due to the constrained boundary set up in our analysis (uptake rate of nutrients defined in SNM3 to 10 mmol/(gDW·h)) and then (−0.46) + (−9.53) = −10 mmol/(gDW·h). Hence, the flux distribution calculated for *S. aureus* was (−0.46/10) = −0.046 mmol/(gDW·h) and that for *D. pigrum* was (−9.53/10) = −0.953 mmol/(gDW·h) because they were a distribution from the confined boundary. Since those new values are relative, we have (−0.046)·(0.5) = −0.023 mmol/(gDW·h) and (−0.953)·(0.5) = −0.4765 mmol/(gDW·h), an overall value of (−0.023) + (−0.4765) = −0.49 mmol/(gDW·h). To re-implement our constrained boundary, we have (−0.49)·(10) = −4.9 mmol/(gDW·h).

We used the same conversion to compare results achieved by setting the abundance of 0.91 for *D. pigrum* and 0.091 for *S. aureus*. Therefore, *D. pigrum* grew 0.21 mmol/(gDW·h) and *S. aureus* grew 3.12 mmol/(gDW·h). We considered one exchange reaction, for instance, EX_leu__L_e. *D. pigrum* consumed 0.09 mmol/(gDW·h) and *S. aureus*, 0.98 mmol/(gDW·h). Hence the exchange shuttle reaction was (−0.98).(0.091) + (−0.09).(0.91) = −0.17 mmol/(gDW·h) (see the **Supplementary CSV Files**).

Our approach with the weight of 0.91 for *D. pigrum* and 0.091 for *S. aureus* achieved the growth of 0.28 mmol/(gDW·h) for *D. pigrum* and 1.47 mmol/(gDW·h) for *S. aureus*, whereas *D. pigrum* consumed 0.46 mmol/(gDW·h) of EX_leu__L_e and *S. aureus*, 0.123 mmol/(gDW·h). Hence, the shuttle exchange of EX_leu__L_e used in the shared environment was (−0.46) + (−0.123) = −0.59 mmol/(gDW·h), which is less than the constrained boundary defined for nutrients in SNM3. (Of note, using the solver in MICOM plays an important role in computation. Using “CPLEX” in MICOM gave the results equal to ours, but MICOM computed no flux distribution. Then, we must have used the “GLPK” solver in MICOM.)

We scaled both growth values achieved to compare our results with results computed by MICOM. This showed that the relative biomass to flux distribution of both approaches was the same as follows: [please refer to the GitHub repository (http://github.com/Biomathsys/DPM-SAU-in-HSA-nose, “optcom_comparison”)]


$$\begin{array}{c} \frac{\text{Growth}\;\text{by}\;\text{MICOM}\;\text{for}\;S.\;aureus}{\text{flux}\;\text{distribution}\;\text{of}\;\text{Ex}\_\text{leu}\_\_\text{L}\_\text{e}\;\text{by}\;\text{MICOM}\;\text{for }S.\;aureus\;}=\frac{3.12}{-0.98}\\ =\frac{1.47}{-0.46}=\frac{\text{Growth}\;\text{by}\;\text{our}\;\text{approach}\;\text{for}\;S.\;aureus}{\text{flux}\;\text{distribution}\;\text{of}\;\text{Ex}\_\text{leu}\_\text{L}\_\text{e}\;\text{by}\;\text{our}\;\text{approach}\;\text{for}\;S.\;aureus\;} \end{array}$$

In contrast, a human nose microbial community tool called NCMW (Glöckler et al., 2022) conveyed the computation since it is consistent with models synthesized in the human nose. We practically fixed all interest settings using a Hydra file available in NCMW. In addition, many GEM models do not grow on the SNM3 due to the lack of some further essential metabolites. Hence, NCMW derived the minimum number of additional metabolites required for the GEM models to grow on SNM3.

To summarize, multiple approaches for building the community and tools broaden the comprehensive viewpoint of community interactions.

### 3.9 Coopm

In the process of pairwise relationship, we also computed COOPM, i.e., a poor medium, using Equation (12). As **Table 2** shows, if the shuttle reactions were defined as either all total exchange reactions or all shared uptake and secretions reactions, the results of using COMPM are the same. Therefore, we applied the MBR of the community achieved for COMPM to compute COOPM. This medium was allowed to obtain only 10% of the community MBR such that removing each metabolite from the set causes no solution. Hence, this computation caused the overgrowth of *S. aureus* while *D. pigrum* does not grow. Due to the faster growth of *S. aureus*, it filled the 10% of the growth required for the community. Therefore, to see how both species on the COOPM as a poor medium could contribute to at least 10% of the MBR on the COMPM, we had to force both species to grow to such an extent to obtain at least 10% of the MBR achieved in COMPM. The COOPM computed is shown in **Table 3**. In this case, both species can survive in this medium while *D. pigrum* is dependent on *S. aureus* for metabolites while *S. aureus* does not require any metabolite from *D. pigrum* in this medium and could grow independently on it. **Table 4** shows the list of exchange reactions enabling *D. pigrum* to obtain metabolites from *S. aureus*. This also made the first result stronger before we tried to enforce both species to grow. *S. aureus* could fill the 10% of community MBR before providing the required metabolites for the growth of *D. pigrum*.

**Table 3:** COOPM computed for *D. pigrum* and *S. aureus*.

Exchange reactions computed in COOPM medium for *D. pigrum *and *S. aureus*		
EX_4abz_e	EX_glc__D_e	EX_ni2_e
EX_26dap__M_e	EX_gly_e	EX_o2_e
EX_ca2_e	EX_k_e	EX_pro__L_e
EX_cl_e	EX_mg2_e	EX_so4_e
EX_cobalt2_e	EX_mn2_e	EX_thm_e
EX_cu2_e	EX_mobd_e	EX_thr__L_e
EX_cys__L_e	EX_na1_e	EX_zn2_e
EX_fe2_e	EX_nac_e	

COOPM is a poor community that allows only 10% of community MBR achieved by COOMPM.

**Table 4:** The consumption and production of metabolites by *S. aureus* and *D. pigrum* in COOPM computed (the flux values are rounded by the threshold *ϵ* = 10^−3^).

	Flux computed using the objective function as a linear combination of growth rates of *D. pigrum* and *S. aureus*		Flux computed using the objective function *α*% MBR*_DP_* + (1 − *α*)% MBR*_SA_*	
Transferred exchange reactions	Flux consumed by *D. pigrum*	Flux produced by *S. aureus*	Flux consumed by *D. pigrum*	Flux produced by *S. aureus*
EX_leu__L_e	−0.0558	0.0558	−0.0170	0.0170
EX_pi_e	−0.109	0.109	−0.033	0.033
EX_ribflv_e	−5.525 × 10^−5^	5.525 × 10^−5^	−1.685 × 10^−5^	1.685 × 10^−5^
EX_arg__L_e	−0.036	0.036	−0.011	0.011
EX_lys__L_e	−0.042	0.042	−0.012	0.012
EX_glu__L_e	−0.077	0.077	−0.023	0.023
EX_his__L_e	−0.011	0.011	−0.003	0.003
EX_ile__L_e	−0.035	0.102	−0.010	0.010
EX_met__L_e	−0.019	0.019	−0.005	0.005
EX_trp__L_e	−0.007	0.007	−0.002	0.002
EX_val__L_e	−0.053	0.053	−0.016	0.016
EX_phe__L_e	−0.040	0.0400	−0.012	0.012

Zero values represent a tiny flux for the associated exchange reactions. This table shows how *S. aureus* and *D. pigrum* interact in a poor community. The list of reaction identifiers in the first column contains the transferred exchange reactions between *D. pigrum* and *S. aureus*.

**Figure 5:**
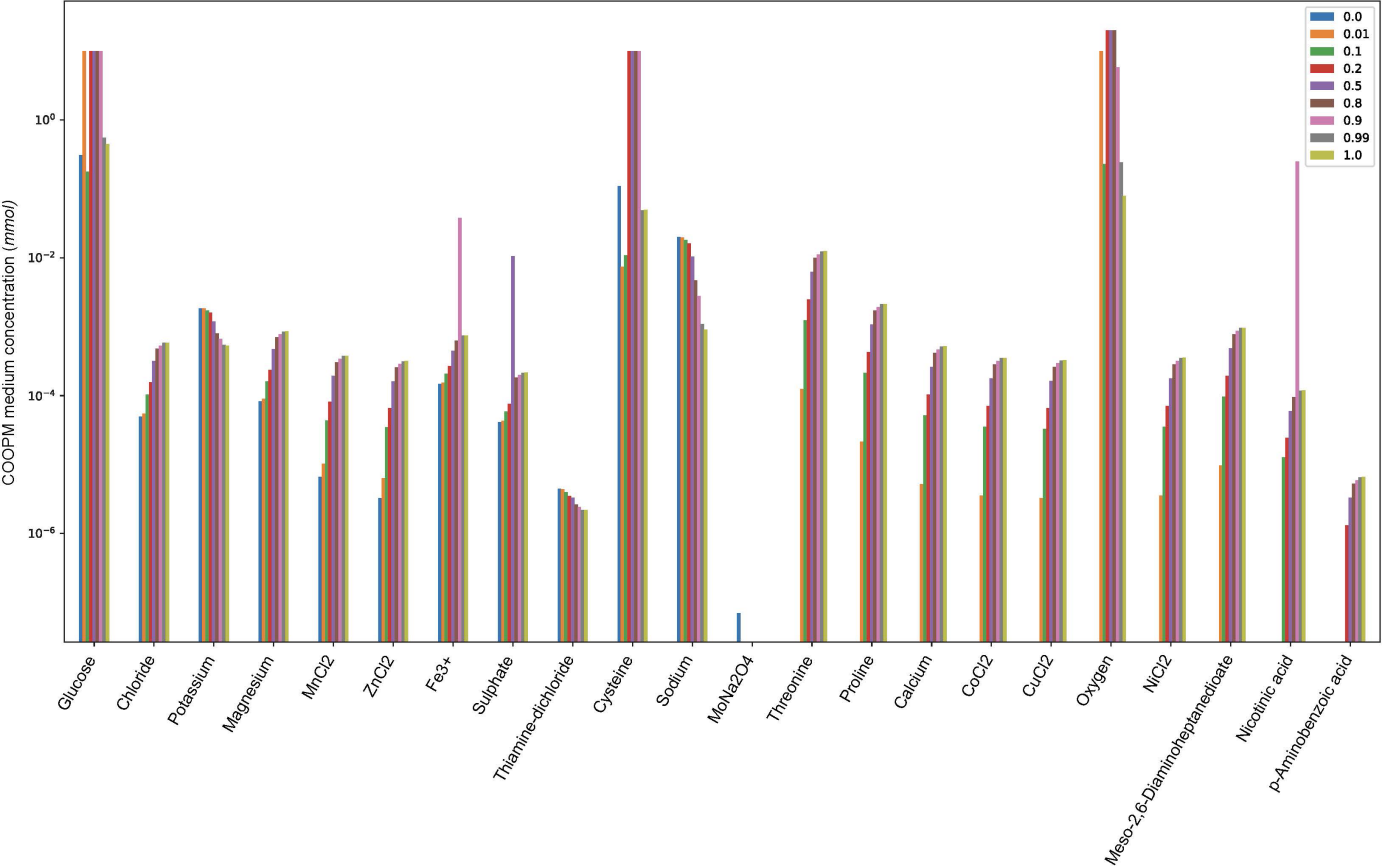
The changes of α ∈ (0, 1) and the required amount of metabolites in COOPM (poor medium) to produce 10% of community MBR as the community objective function is defined by *α*% MBR*_DP_* + (1 − *α*)% MBR*_SA_***.** *α* = 0 and *α* = 1 mean that the optimization function is the maximization of *S. aureus* and *D. pigrum*, respectively. For instance, if we look at threonine, we do not see any blue color, which was indicated for *α* = 0. This means that if the objective function in the community with poor nutrients is defined only by the maximization of *S. aureus*, the change of this metabolite does not play an important role.

The results represented for computation of COOPM did not show any difference in using either the linear combination of growth functions of *D. pigrum* and *S. aureus* or the convex objective function as *α*% MBR*_DP_* + (1−*α*)% MBR*_SA_* for *α* ∈ (0, 1). However, when we applied the convex objective function, we could see how the change of *α* ∈ (0, 1) indicates the required amount of metabolites of COOPM (poor medium) to produce 10% of community MBR as shown in **Figure 5**. This figure also showed the relation between different weights to species by *α* and the usage of metabolites of COOPM (whether less or more).

**Tables 3**, **4** indicate the COOPM regardless of the type of objective function except for one exchange reaction, which was highlighted in the tables. They also showed that in the poor medium, *S. aureus* dominates the community by using nutrients to grow at the expense of *D. pigrum*.

**Figure 6:**
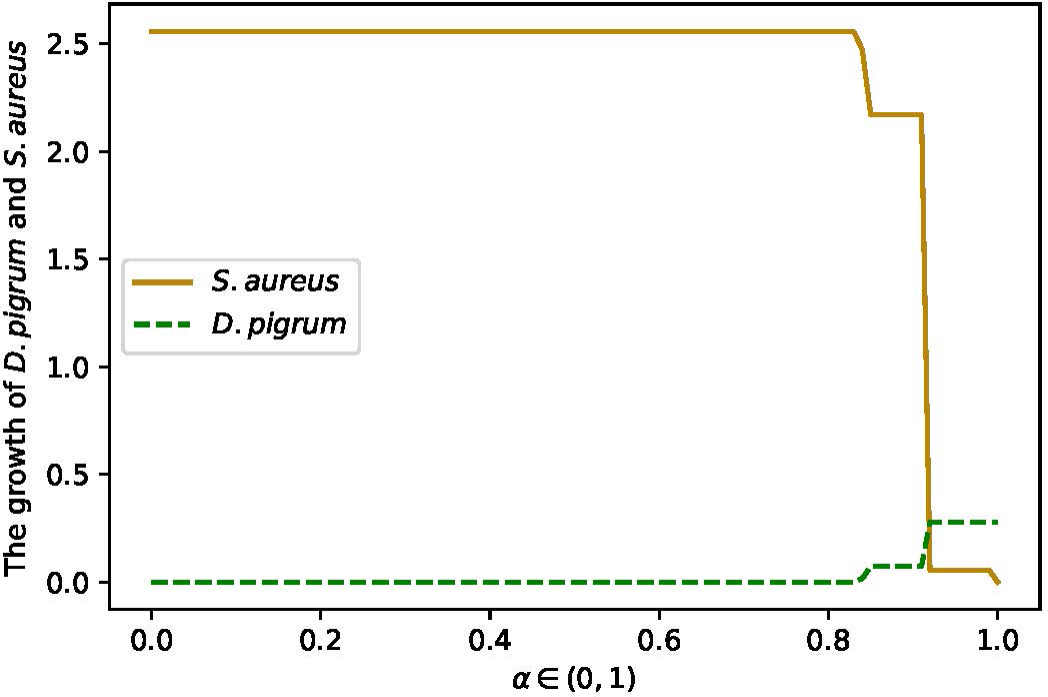
The changes in growth rate of *D. pigrum* and *S. aureus* in respect to *α* defined in the community objective function *α*% MBR*_DP_* + (1 − *α*)% MBR*_SA_* in COMPM.

As an assurance, we continued the computation of COOPM with an additional setting. We applied the compartmentalized approach with shuttle reactions as these were defined by the COMPM medium. In this new setting, both weighted linear and convex objective functions were implemented. When the convex objective function *α*% MBR*_DP_* + (1 − *α*)% MBR*_SA_* for *α* ∈ (0, 1) was applied, as **Figure 6** shows, *α* = 0.85 balanced the growth rates of *D. pigrum* and *S. aureus.*

**Figure 7:**
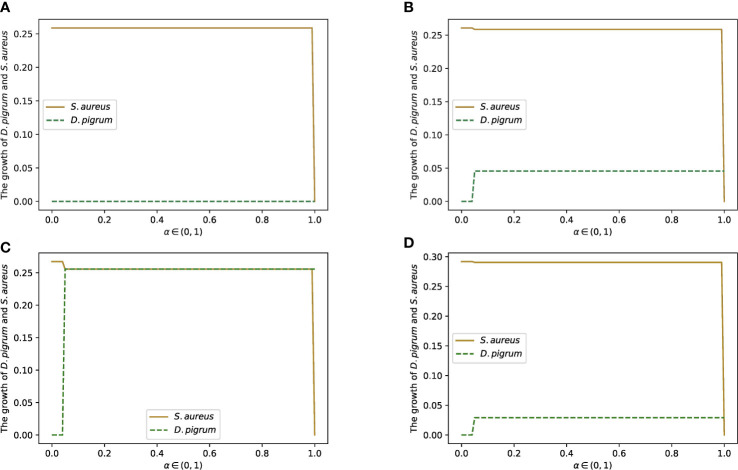
The change in the growth rates of *D. pigrum* and *S. aureus* in respect to *α* defined in the community objective function *α*% MBR*_DP_* + (1 − *α*)% MBR*_SA_* as they are in the community with the COOPM medium. COOPM is a poor medium in such a way that it allows the species to produce only 10% of MBR achieved in COMPM. This shows that if we do not enforce species to fill the 10% of MBR, then *S. aureus* always dominates. However, the enforcement of survival for both species provides the condition for *D. pigrum* to grow since *D. pigrum* depends on the nutrients produced by *S. aureus*. This could be compensated if the weight of *S. aureus* was not less than *D. pigrum*. **(A)** Is implemented with the weights of 0.85 for *D. pigrum* and 0.15 for *S. aureus* in COMPM to compute the community MBR as the enforcement for the survival of both species was not applied. Therefore, *S. aureus* dominates. **(B)** Is implemented with the weights of 0.85 for *D. pigrum* and 0.15 for *S. aureus* in COMPM to compute the community MBR as the enforcement for the survival of both species was applied. However, *D. pigrum* still has to wait for *S. aureus* to grow. **(C)** Is implemented with the weights 1:1 for *D. pigrum*, and *S. aureus* in COMPM to compute the community MBR as the enforcement for the survival of both species was applied. Therefore, using the high weight for *S. aureus* caused the production of the required nutrients for *D. pigrum* and resulted in the growth of *D. pigrum*. **(D)** Is implemented with the weights 10:1 for *D. pigrum* and *S. aureus* in COMPM to compute the community MBR as the enforcement for the survival of both species was applied. However, *D. pigrum* still has to wait for *S. aureus* to grow.

Therefore, *α* = 0.85, i.e., a weight of *α* = 0.85% to *D. pigrum* and one of (1 − *α*) = 0.15% to *S. aureus*, was applied. The objective function *α*% MBR*_DP_* + (1 − *α*)% MBR*_SA_* for COMPM was computed. In the end, COOPM was calculated in a way that the community could reach 10% of MBR achieved by COMPM. The results have also followed **Tables 3**, **4**. Furthermore, the interesting results were the change in the growth rate of species in respect to *α* on COOPM, as shown in **Figures 7A, B** when *α* = 0.85 was used.

Furthermore, the other weights were also implemented to confirm the results. For instance, weight 1:1 for *D. pigrum*: *S. aureus* in the computation of community MBR on COMPM was used. Thus, the achievement of 10% of this MBR as a goal was settled to compute the COOPM medium. In addition, the setting was made in a way to ensure that both species survive. Therefore, it was observed in **Figure 7C** that *D. pigrum* could grow approximately as much as *S. aureus* in COOPM with poor nutrients. It happened because the higher weight of *S. aureus* made the environment richer in nutrients required for the growth of *D. pigrum*. This was also approved when weight 10:1 for *D. pigrum*: *S. aureus* in the computation of community MBR on COMPM was used, and then the COOPM medium was computed as indicated in **Figure 7D**.

Therefore, regardless of the approach implemented for the calculation of COOPM, *D. pigrum* depends on *S. aureus* for metabolites in the poor medium, and *S. aureus* displays a faster growth. This means that *S. aureus* uses all nutrients available in the poor medium to grow, but it does not reach its MBR. Therefore, it cannot provide sufficient nutrients to *D. pigrum*, which fails to grow. Only enforcement of *D. pigrum* by using a higher weight for *D. pigrum* than *S. aureus* allows *D. pigrum* to grow, a condition that *in vitro* can be achieved by starting the co-culture with different initial optical densities. However, this is far from being physiologically relevant and leads to the unsolved question: how is it possible in nature to give a higher weight to *D. pigrum* than *S. aureus* in a community with poor nutrients?

### 3.10 Robustness analysis

**Figure 8:**
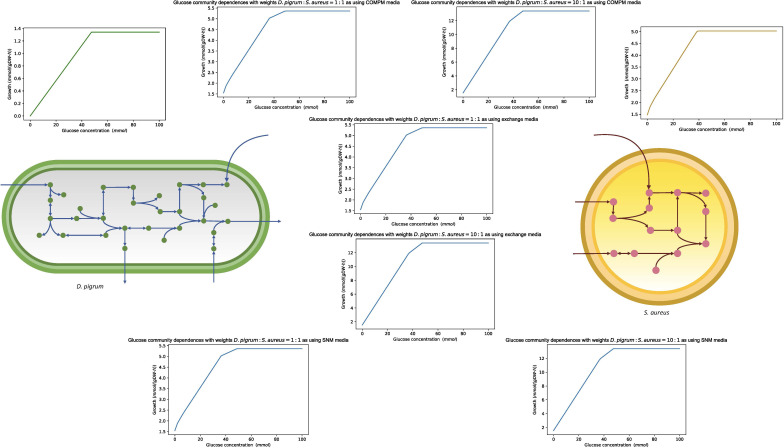
The figure with the green curve shows the linear dependency of glucose concentration in a single model of *D. pigrum*. In contrast, the one with gold color represents the slight dependency of the glucose concentration of *S. aureus* as it can grow even though the glucose concentration reaches zero. Each blue curve shows the glucose dependency in the community with all different media. We used a compartmentalized approach with shuttle reaction. Different weights were also applied.

Robustness is defined as the property of the system conferring tolerance against those perturbations that might affect the system’s functional body. Despite its fundamental importance, robustness is hard to quantify. With respect to metabolic networks, robustness is a measure of the change in the maximal flux of the objective function when the optimal flux through any particular metabolic reaction is changed. Here, we determined the robustness characteristics of the metabolic networks of *D. pigrum, S. aureus*, and the community of both species to flux changes in the essential enzymatic reactions. The essential enzymes (for growth on glucose-minimal medium) were previously identified through an *in silico* analysis in **Figure 8**. We calculated the effect of the change of the flux vector on the maximal growth. The flux through the exchange reaction of interest was reduced in the range of (−10, 0), and the objective function was calculated. All calculations have been done on the metabolic network of *D. pigrum, S. aureus*, and the community of both species. This helped to distinguish how one species behaves in isolation compared with being in a community and allowed the identification of individual cooperative and antagonistic traits. We focused on some interesting compounds. As shown in **Figure 8**, *D. pigrum* was strictly glucose-dependent and could not grow without glucose, while *S. aureus* was loosely glucose-dependent; i.e., it could grow without glucose. In the community, as the higher weight was used for *D. pigrum*, the community’s growth rate was increased as the glucose concentration changed.

**Figure 9:**
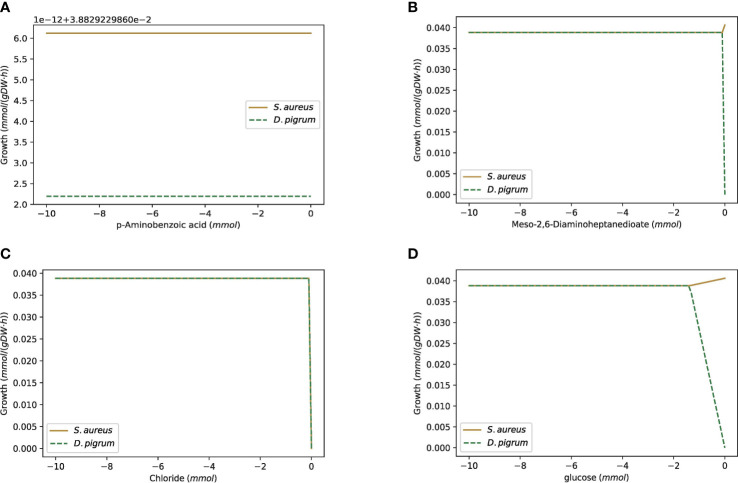
The robustness analysis of the COOPM community of *D. pigrum* and *S. aureus* with respect to alterations of the flux levels of all essential metabolic reactions included in the community. The gold line represents the growth of *S. aureus* and the green one is for *D. pigrum*. **(A)** The change of 4 aminobenzoate exchange. The changes of MoO_4_^–2^ (molybdate) and thiamin exchanges follow the same plot. **(B)** The change of the meso-2,6-diaminoheptanedioate exchange. The changes of Ca^2+^, Co^2+^, Cu^2+^, Ni^2+^, L-Proline, and L-threonine follow the same plot. **(C)** The change of the Cl^–^ exchange. The changes of Fe^2+^, K^+^, Mg^2+^, Mn^2+^, Na^+^, Zn^2+^, Nicotinate, O_2_, ${\mathrm{SO}}_{4}^{2-}$ (sulfate), and L-cysteine exchange follow the same plot. **(D)** The change of the D-glucose exchange.

**Figure 9** shows the robustness characteristics of the COOPM community of *D. pigrum* and *S. aureus* with respect to alterations of the flux levels of all essential metabolic reactions available in the community when the objective function in the community was defined as a weighted linear combination of both species’ growth.

As **Figure 9D** shows, *D. pigrum* was also glucose-dependent in the poor community, while *S. aureus* could still grow even without glucose.

### 3.11 Prediction of interaction between *D. pigrum* and *S. aureus* based on computed communities COMPM and COOPM

We applied Equations (13) and (14) to computationally predict the interaction between *D. pigrum* and *S. aureus* and compare our results with experimental ones. As **Table 5** shows, the results attained through calculation of formula were similar to our prediction through analysis of the behavior of species in the community. When weight 1:1 for *D. pigrum*: *S. aureus* was applied, the failure of growth for *D. pigrum* in the community on COMPM showed that *S. aureus* hardly competed with *D. pigrum*. Since *S. aureus* had a faster and higher growth, *D. pigrum* failed. On COOPM, even though we forced both species to survive, they could not cooperate. As **Figure 7C** showed, they both grew on COOPM since *S. aureus* already reached its maximum growth on COMPM and thus could be capable of producing the required nutrients for the growth of *D. pigrum.* The cooperation interaction could be seen when weight 10:1 for *D. pigrum*: *S. aureus* was applied or by giving more contribution to *D. pigrum* in the community as shown in **Figures 7B, D**.

**Table 5:** The prediction of interaction between species *D. pigrum* and *S. aureus* using Equations (13) and (14).

Weight 1:1	PCMS	1.0	Maximal competition
	PCPS	0.0	No cooperation
Weight 10:1	PCMS	−9.0	Cooperation
	PCPS	0.9	Cooperation

When weight 1:1 for *D. pigrum*: *S. aureus* was used, the computation of COMPM and COOPM showed maximal competition and no cooperation. As weight 10:1 for *D. pigrum*: *S. aureus* was applied, the species started to cooperate with each other.

### 3.12 How does the community provide the metabolites necessary for the growth of the single species?

Genome-scale modeling often considers species in isolation. However, this approach is oversimplified as it creates a situation that rarely occurs in nature. In real-life environments, species usually thrive in complex communities in which the growth of a single species depends on the interactions with other species in the population. To analyze how *D. pigrum* and *S. aureus* influence each other’s growth, we considered the GEM of *D. pigrum* in the absence of amino acids L-isoleucine and L-methionine, as well as the metabolite 2,6-diaminoheptanedioate, a condition that does not allow *D. pigrum* to grow on SNM3 in isolation. We applied the custom model with SNM3 medium on these two species one more time. In addition, we ensured both species reach at least 10% of the MBR of the whole community. Furthermore, we added one additional constraint to our custom model to allow the exchange of L-isoleucine and L-methionine between *D. pigrum* and *S. aureus*, as these metabolites are among the exchange reactions of *S. aureus*. The third metabolite required for growth of *D. pigrum*, 2,6-diaminoheptanedioate, was manually added to the custom model since *S. aureus* is also unable to provide it. This means that either the community might need another partner species that produces this metabolite in nature or there is a clear limitation of the modeling since 2,6-diaminoheptanedioate is an essential component of the bacterial cell wall. Hence, it is released in the environment upon bacterial cell lysis, thus becoming available for uptake by *D. pigrum.*

**Figure 10:**
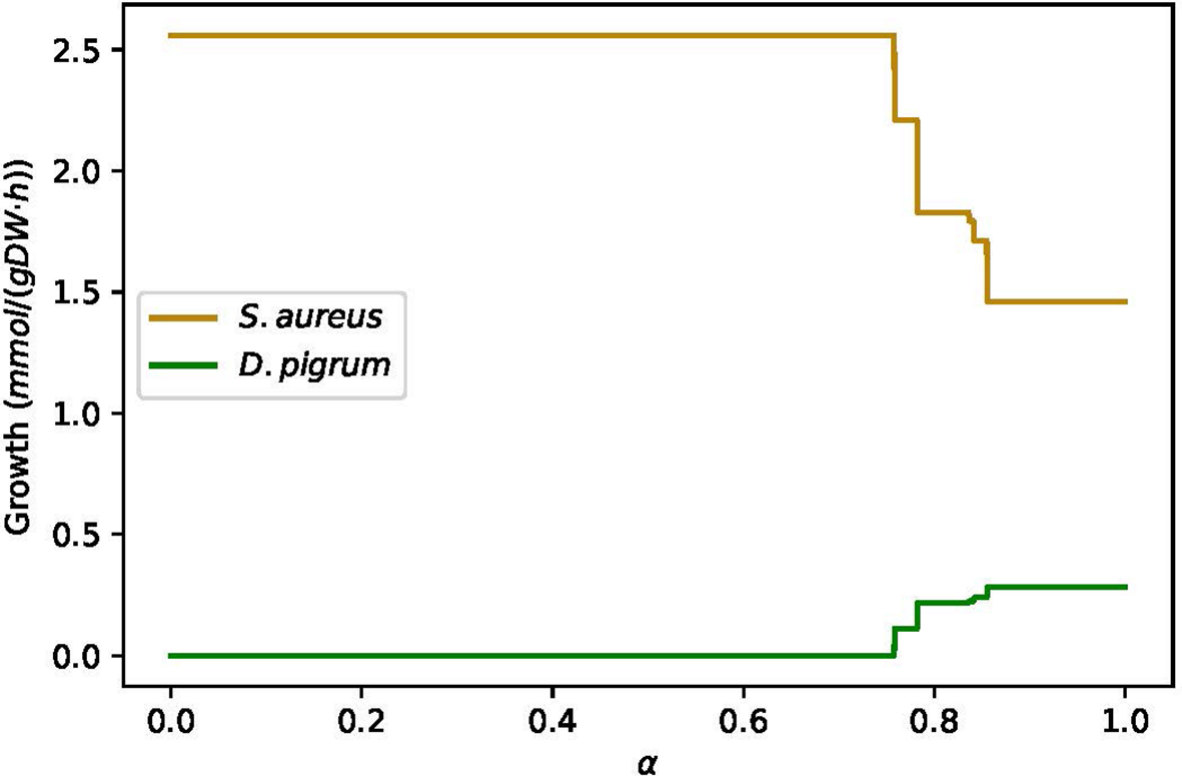
The *D. pigrum* is used in the absence of amino acids L-isoleucine and L-methionine, as well as metabolite 2,6-diaminoheptanedioate while *S. aureus* grows on SNM3. The figure represents by which factor of *α S. aureus* provides the necessary metabolites for *D. pigrum* to allow it to grow. The community objective function is defined as *α*% MBR*_DP_* + (1 − *α*)% MBR*_SA_* and the medium is assumed to be SNM3.

**Figure 11:**
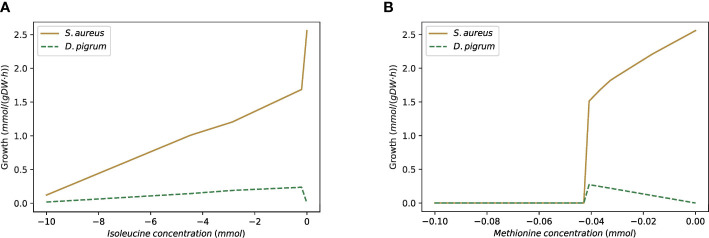
The custom model with SNM3 medium is used as *D. pigrum* appears in the absence of L-isoleucine and L-methionine and then without growth on SNM3. *S. aureus* provides both necessary amino acids for *D. pigrum*. **(A)** Shows the change of L-isoleucine in the community while (*D. pigrum * has no growth. **(B)** Shows the change of L-methionine in the community while *D. pigrum* has no growth.

In this case, if we use the equal weights for both species, then *S. aureus* reaches its MBR while *D. pigrum* fails to grow. Hence, we applied the objective function as *α*% MBR*_DP_* + (1 − *α*)% MBR*_SA_* to see when the growth for *D. pigrum* is feasible. As **Figure 10** shows, we observed roughly close to *α* = 0.8 for *D. pigrum*; *D. pigrum* starts growing. Moreover, we could observe how *S. aureus* provides L-isoleucine and L-methionine for *D. pigrum* (**Figure 11**).

Therefore, we used the weight of 0.85 for *D. pigrum* and 0.15 for *S. aureus*. **Table 6** shows the shared metabolites in this setting. In this condition, we observed growth of *D. pigrum* within the community, indicating that *S. aureus* can provide some of the metabolites that *D. pigrum* needs for growth. Hence, this type of analysis allows inferring which species live in a community in nature and which one can also live in isolation.

**Table 6:** The consumption and production of metabolites by *S. aureus* and *D. pigrum* in COOPM are computed in that way; *D. pigrum* has no growth in isolation (the flux values are rounded by the threshold *ϵ* = 10^−3^).

Flux computed using the objective function *α*% MBR*_DP_* + (1 − *α*)% MBR*_SA_*		
Transferred exchange reactions	Flux consumed by *D. pigrum*	Flux consumed by *D. pigrum*
EX_ile__L_e	−0.079	0.079
EX_met__L_e	−0.042	0.042

The first column gives the list of transferred exchange reactions between *D. pigrum* and *S. aureus* in COOPM.

Herein, we applied three optimization approaches to the community and compared the results attained by all approaches. The three optimization problems we used based on integrated, pooled, and shuttle reactions are multi-objective optimization problems. In the process of finding the solution for these problems, two stages can be considered. One stage can be the optimization of the objective functions, and the other is deciding what kind of trade-offs are appropriate from the decision-maker perspective. The decision-maker—the designer—determines the set of optimal compromise solutions that have to be identified by an effective and complete search procedure to carry out the best choice (Chiandussi et al., 2012). Therefore, these techniques are discussed by analyzing their advantages and disadvantages.

Priori technique: Takes decisions before searching and includes those approaches that assume that either a certain desired achievable goal or a certain pre-ordering of the objectives can be performed by the decision-maker prior to the search. This approach was herein used in the pooled approach. The main advantage of this method is its simplicity and effectiveness because it reduces the computational cost. However, its main disadvantage is the definition of user-specified minimum growth rate and user-specified trade-off factor. For this reason, this method fits especially well when the ideal value of the objective functions is known and can be set as a target.

Posteriori technique: Searches before making decisions and does not require prior preference information from the decision-maker. Some of the techniques included in this category are among the oldest multi-objective optimization approaches proposed. This approach is used by optimization created in a defined integrated approach. This method’s most important benefits are its simplicity and efficiency. Its main disadvantage is the difficulty of determining the appropriate weight coefficients to be used when enough information about the problem is not available. This can be improved by the usage of different portions of weight coefficients. OptCom and MICOM are both Posteriori techniques. OptCom uses a solution from a multi-objective optimization (Pareto front) using bi-linear optimization, while MICOM uses L2 regularization. Both do not use any information from the decision-maker. This approach was herein used by optimization created in shuttle reaction while either we ensured both species survive or tried to run it for different weights to find the best possible solution. Therefore, the multi-objective optimization of each species’ objective appeared. Moreover, we applied the MICOM package using the OptCom community objective. In order to make MICOM work for our purpose, which is analyzing the nasal microbial community, we modified MICOM. After this modification, the results showed that despite different scaling, *D. pigrum* grew if the abundance was 10 times higher than *S. aureus.*

Progressive technique: Integrates search and decision-making. Usually, it finds a non-dominated solution, then gets the reaction of the decision-maker regarding this non-dominated solution and modifies the preferences of the objectives accordingly, and repeats the two previous steps until the decision-maker is satisfied or no further improvement is possible. This technique was used in an integrated approach while optimizing the community, and each species was ranked equally. This approach seems particularly suitable to solve multi-objective optimization problems because it deals simultaneously with a set of possible solutions. The main disadvantage is the computational cost that is, in general, very high due to the operational process of the method itself.

All the approaches above capture some of the important features of the nasal community and convey one specific way of analyzing the community, which are summarized as follows:

Increases in nutrient concentrations associated with media definitions have led to changes in the growth and secreted metabolites that have become clear. Therefore, there is an action such as the presence/absence of nutrients (based on media definition) and the growth of *S. aureus* with producing some secreted metabolites. At some later time, there is a consequence such as the growth of *D. pigrum* by taking up secreted metabolites of *S. aureus* and producing new compounds, which lead to antimicrobial effects. This can confuse the community’s interpretation, called time lag, and can separate the cause and effect. This time lag between when a community of *S. aureus* and *D. pigrum* was formed, when they reached maximum biomass, and when they showed their natural interaction happened with dependency on species’ mortality and growth rates as well as the dependency on the other species available in the community.

## 4 Discussion

With the challenge implemented in this study, we can assume that the relationship between species can sometimes be time-lagged; for example, if one species increases its abundance at a specific moment, another species might only disappear late, as we also noticed that the interaction between *D. pigrum* and *S. aureus* changed. Furthermore, competition can happen in two modes, called interference and exploitation. Bacteria engaging in exploitation competition compete by preventing their competitors from accessing resources by either rapidly consuming or sequestering these supplies as *S. aureus* behave against *D. pigrum* while they were in the COOPM with the availability of fewer nutrients. In contrast, bacteria using interference competition produce toxic effectors to directly inhibit their competitors, as happened when *D. pigrum* reached its maximum biomass rate in the community with *S. aureus*. Therefore, approaches, objective functions, tolerance defined in optimization, shared environment, and computational methods such as optimization solvers played an essential role in the community metabolic analysis. Neither one approach nor one kind of objective function can, in general, be a solution for any community construction. A combination of approaches, methods, structures, and biological knowledge can be practical. In our community, each approach directed us to particular results. For instance, we noticed that *D. pigrum* did not grow in SNM3 in isolation. This required some metabolites, which *S. aureus* could have provided when they were in the community. However, one could criticize this statement if one considered other species in the community. This is likely that other species might have provided the essential amino acids for the growth of *D. pigrum*. Hence, *D. pigrum* started to grow where either *S. aureus* was available or other species might have provided the essential metabolites for the growth of *D. pigrum* without any dependency on *S. aureus* at the first step. In the next step, when *D. pigrum* reached its maximal biomass rate while in the community, it released D-serine, and the antibacterial role of *D. pigrum* started. Indeed, species isolated from the human nasal community are well-known to use specialized (secondary) metabolites with antimicrobial properties to engage in interference competition. This was shown in the COMPM and computation of the maximal biomass rate of the compartmentalized community. To give more weight to the growth rate of *D. pigrum*, this role became stronger and stronger. Therefore, we hypothesized that *D. pigrum* is a mutualist concerning its human host rather than a purely commensal bacterium. Commensalism and mutualism are the symbiotic ways organisms interact with each other with differing degrees of benefit. Mutualism is defined as any interaction between two species that benefits both. However, commensalism is a sharing of the same environment by two organisms where one species benefits and the other is unaffected. By forcing the growth of *D. pigrum* with defining the higher weight in COOPM, *D. pigrum* grew while *S. aureus* has provided some required metabolites for the growth of *D. pigrum*. This showed the dependency of *D. pigrum* to *S. aureus* in some required metabolites for the growth.

Therefore, the interactions strongly depend on what is present/absent in the community environment, and microbial community members cannot preserve their role the whole time. They switched their roles. This needs dynamic analysis. The dynamic metabolic community where species can change their metabolic behavior during the experiment is closer to natural environments. Herein, FBA is used to study the metabolic flux at a particular steady state of the system. A famous statement says, “Friends become enemies and vice versa”, secreting/consuming different substances on different timescales would be sufficient to change interaction signs over the course of time. Since our study focused on CBM, not the dynamic behavior of metabolic species over time, by applying different media definitions, changing the community environment over time is taken into account. By those definitions, we can observe that species show different behavior against each other in the community regarding the availability of essential metabolites that alter over time. Additionally, our results explain that in the beginning, these two species benefit from each other, which helps one grow increasingly, which highlights the presence of events in the past that have had effects on the current state. However, after a certain point, the friend becomes an enemy and inhibits the other one. Therefore, this explanation points out the way forward for incorporating time lag effects into predictions of future states. Furthermore, it shows that if we want to benefit from the inhibition of pathogens, which conditions should be considered.

As already identified, *D. pigrum* is a member of the lactic acid bacteria (LAB) and, as other LAB, is more frequently found in the URT of healthy individuals than in the UTR of subjects affected by chronic respiratory diseases (Laufer et al., 2011; Biesbroek et al., 2014; De Boeck et al., 2019; De Boeck et al., 2021). Accordingly, *D. pigrum* and *S. aureus* are often inversely correlated in the adult nasal microbiota (Liu et al., 2015; Escapa et al., 2018). Brugger et al. (2020) also showed that *D. pigrum* inhibits *S. aureus* growth *in vitro*. The authors predicted that the production of L-lactic acid by *D. pigrum* in a rich medium is unlikely to account for the negative associations with *S. aureus* (Brugger et al., 2020) since the human nose environment is not a rich-nutrient milieu and L-lactic acid is unlikely to be produced by *D. pigrum in vivo*. By mining the genome of *D. pigrum*, Brugger et al., 2020 speculated possible alternative mechanisms behind the inhibitory effect on *S. aureus*, including nutrient competition and secretion of anti*-S. aureus* secondary metabolites. In fact, the authors identified a diverse repertoire of biosynthetic gene clusters (BGCs) in the genomes of 11 *D. pigrum* strains. These BGCs were predicted to encode bacteriocins, including lanthipeptides, which could play a role in niche competition (Brugger et al., 2020). Lanthipeptides are ribosomally synthesized and post-translationally modified antimicrobial peptides formed by dehydration of serine/threonine residues conjugated to cysteine (Arnison et al., 2013). Interestingly, our modeling data indicate that when in community with *S. aureus* (see the **Supplementary CSV Files**), *D. pigrum* catalyzes the following chemical reaction with the enzyme D-serine ammonia-lyase (EC 4.3.1.18):


D-serine⇋pyruvate+ammonia

**Figure 12:**
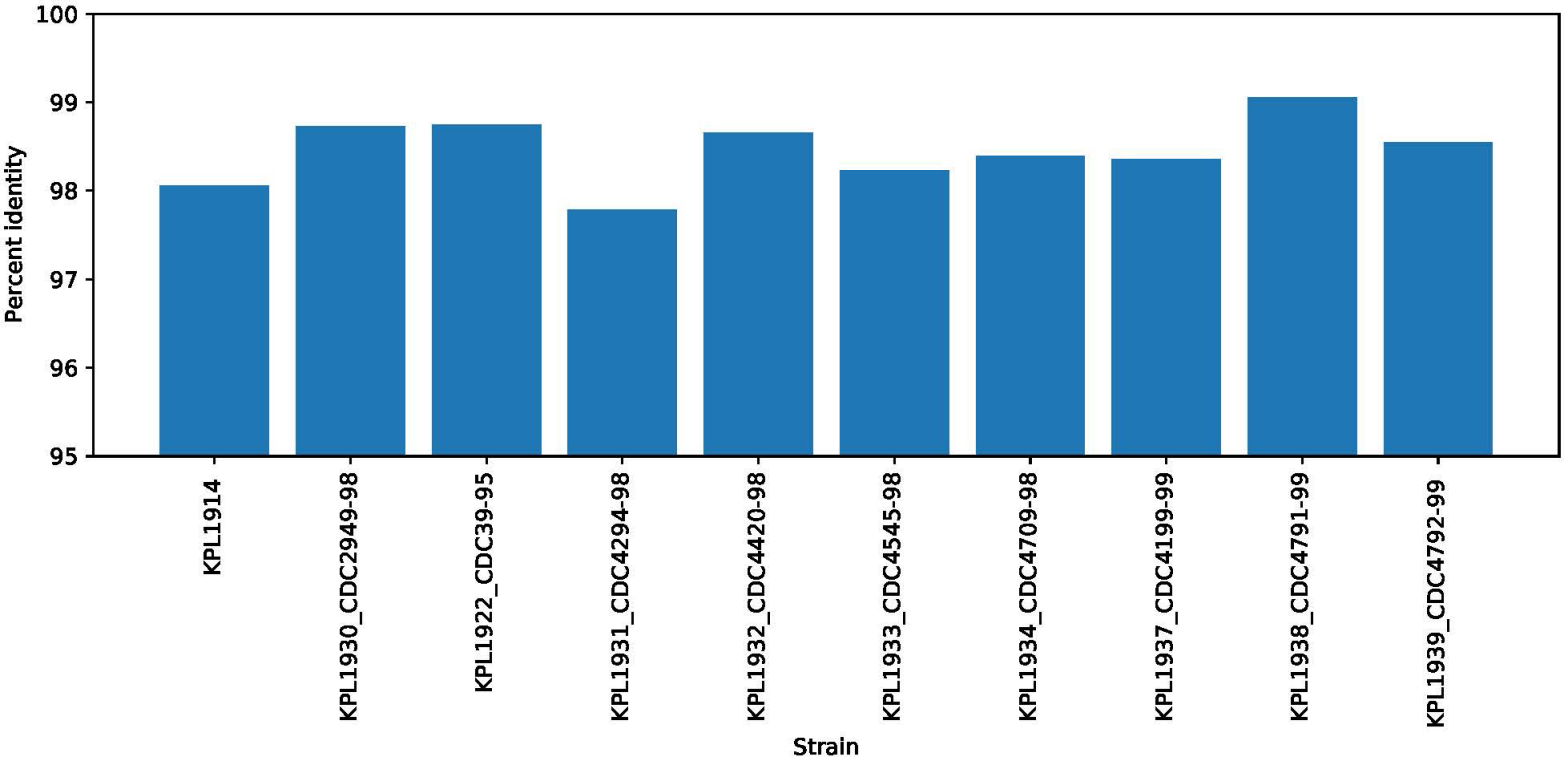
The figure represents the similarity between *D. pigrum* strain VPs-KB5 applied in this study and strains surveyed by Brugger et al. assessed by BLAST. As the percent identity shows, our study’s strain has high similarity to the strains studied by Brugger et al, 2020.

It can also take up cysteine, an amino acid available in the human nose environment, suggesting that the *D. pigrum* strain used for our community model has the potential to produce and deploy lanthipeptides against *S. aureus*. Of note, we found a high level of similarity between our *D. pigrum* strain and the strains surveyed by Brugger et al., 2020 as assessed by a Basic Local Alignment Search Tool (BLAST) analysis (Altschul et al., 1990). The results can be found in **Figure 12**.

In summary, we speculate that, as in our simulation in poor medium, *D. pigrum* should barely grow in the human nose environment while *S. aureus* should grow less than its MBR. However, if the initial density of *D. pigrum* is higher than that of *S. aureus*, the former should be able to grow at the expense of the latter. In our COOPM community model, to allow growth of *D. pigrum*, we had to manually add 2,6-diaminoheptanedioate, an essential component of the bacterial cell wall (Zühlke et al., 2016; Li et al., 2019), on the assumption that *S. aureus* cannot provide it through an exchange reaction. This is a limitation of the model because 2,6-diaminoheptanedioate is released in the environment upon bacterial cell lysis, thus becoming available for uptake by *D. pigrum*. Hence, it is tempting to speculate that *in vivo D. pigrum* might foster the release of metabolites such as 2,6-diaminoheptanedioate by secreting bacteriocins and triggering the death of the surrounding bacterial cells, such as *S. aureus*. The ability of *D. pigrum* to grow at the expense of *S. aureus*, as observed in our model and in the *in vitro* assay by Brugger et al., 2020, might explain why the presence of *D. pigrum* in the nasal microbiota is often negatively correlated with that of *S. aureus*. It also suggests that, when administered as a probiotic, i.e., in relatively large amounts, *D. pigrum* should be able to displace *S. aureus* from the nasal microbiota of carriers effectively.

Therefore, our analysis supports the idea that microbe-targeted interventions may reshape the composition of nasal microbial communities and suggests that metabolic interactions may be key drivers of community structure. As such, our work sets the premises for future experimental studies aimed at investigating the molecular basis of interference between *D. pigrum* and *S. aureus*, as well as the therapeutic potential of *D. pigrum* as a nasal probiotic to displace *S. aureus* from the microbiota. This will also raise the requirement of not only long-term data but also very-long-term data with a simulation model that can be combined because time lags still remain one of the most significant challenges to developing a framework for forecasting future species interaction throughout the community.

## Data availability statement

The community model of *D. pigrum* and *S. aureus* is available in the BioModels Database (Malik-Sheriff et al., 2020) as an SBML Level 3 Version 1 file (Bergmann et al., 2018) within a COMBINE Archive OMEX file (Bergmann et al., 2014) at https://www.ebi.ac.uk/biomodels/models, under the accession number MODEL2209060002. The source code is freely available at http://github.com/Biomathsys/DPM-SAU-in-HSA-nose. The package depends on third-party software that was successfully tested using Python (version 3.7 or later), COBRApy (version 0.19.0 or later), CPLEX (version 20.1.0), and operating systems macOS Big Sur (version 11.5.1), Windows (version 10), and Linux (distribution Ubuntu, version 20.10 or later). Alternativiely, other solvers for linear programing may be used as long as those are supported by COBRApy.

## Author contributions

RM and AD developed the conceptual idea. RM derived the mathematical method. MG implemented the method under the guidance of RM. RM and MG performed the analysis and wrote the manuscript. AD supervised the work and critically revised the manuscript and the figures. All authors contributed to the article and approved the submitted version.

## Funding

This research was funded by the German Center for Infection Research (DZIF, doi: 10.13039/100009139) within the *Deutsche Zentren der Gesundheitsforschung* (BMBF-DZG, German Centers for Health Research of the Federal Ministry of Education and Research) grant No. 8020708703 and supported by infrastructural funding from the *Deutsche Forschungsgemeinschaft* (DFG, German Research Foundation), Cluster of Excellence EXC 2124 Controlling Microbes to Fight Infections. Parts of this work were funded by the Federal Ministry of Education and Research (BMBF, Germany) and the Baden-Württemberg Ministry of Science as part of the Excellence Strategy of the German Federal and State Governments. The authors acknowledge support from the Open Access Publishing Fund of the University of Tübingen (https://uni-tuebingen.de/de/58988).

## Acknowledgments

The authors thank Libera Lo Presti for her valuable and critical feedback on this manuscript.

## Conflict of interest

The authors declare that the research was conducted in the absence of any commercial or financial relationships that could be construed as a potential conflict of interest.

## Publisher’s note

All claims expressed in this article are solely those of the authors and do not necessarily represent those of their affiliated organizations, or those of the publisher, the editors and the reviewers. Any product that may be evaluated in this article, or claim that may be made by its manufacturer, is not guaranteed or endorsed by the publisher.
