# Supplementary figures and images for: Towards the human nasal microbiome: Simulating D. pigrum and S. aureus
Source: Front Cell Infect Microbiol. 2022 Oct 11;12:925215. doi: 10.3389/fcimb.2022.925215 (PMC9810029; doi:10.3389/fcimb.2022.925215)

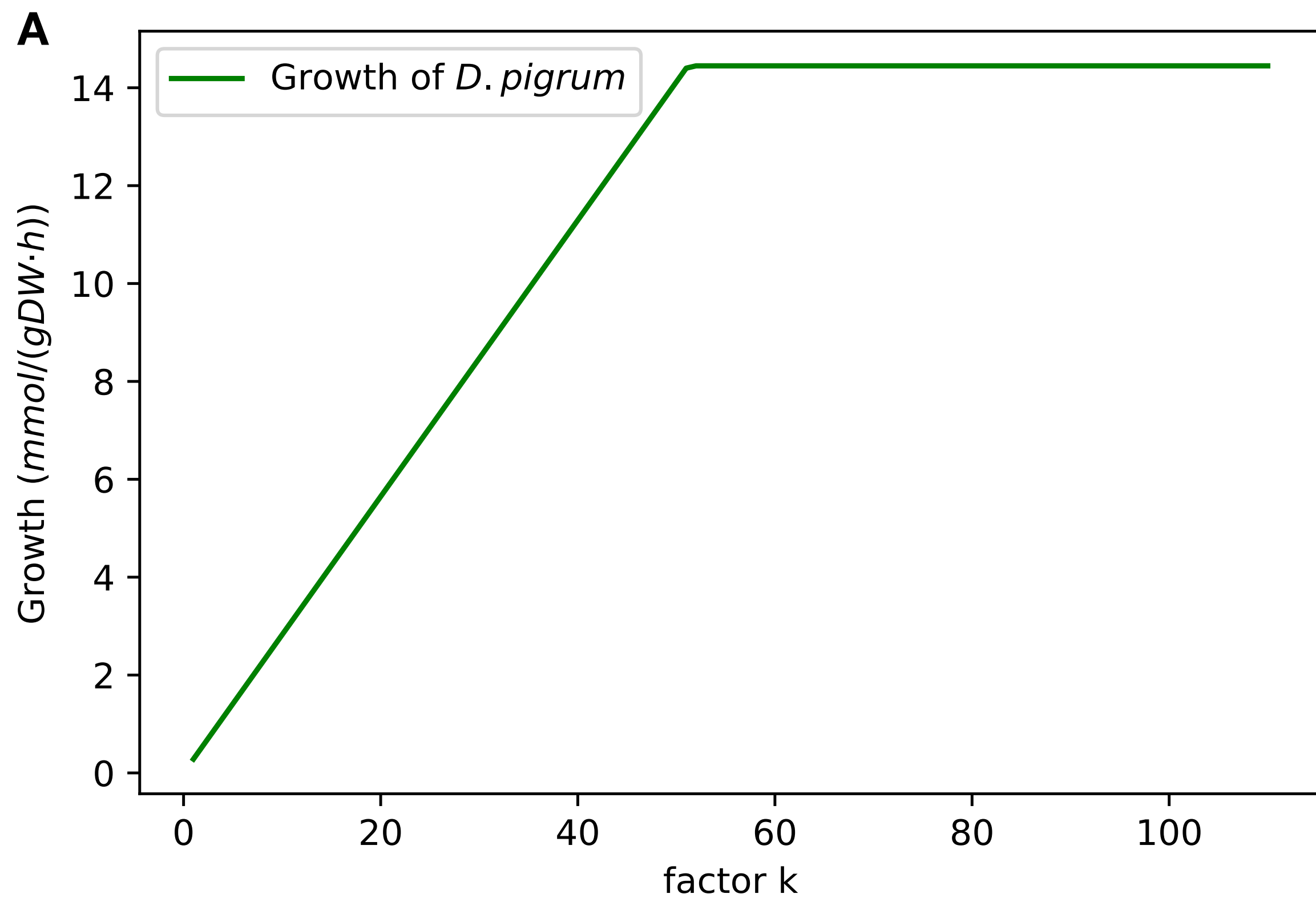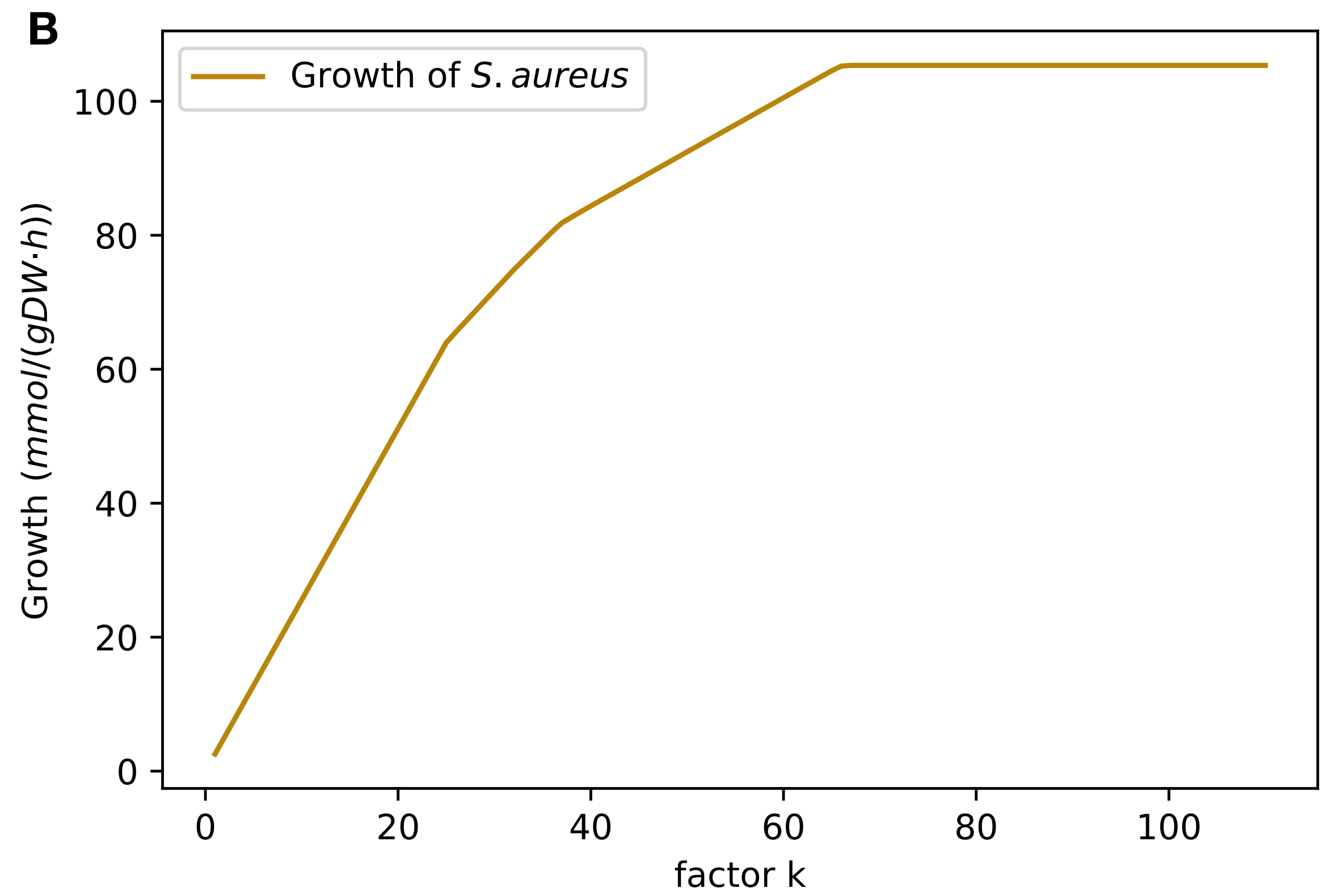

Supplement: Supplementary file 1 [file DataSheet_1.pdf]

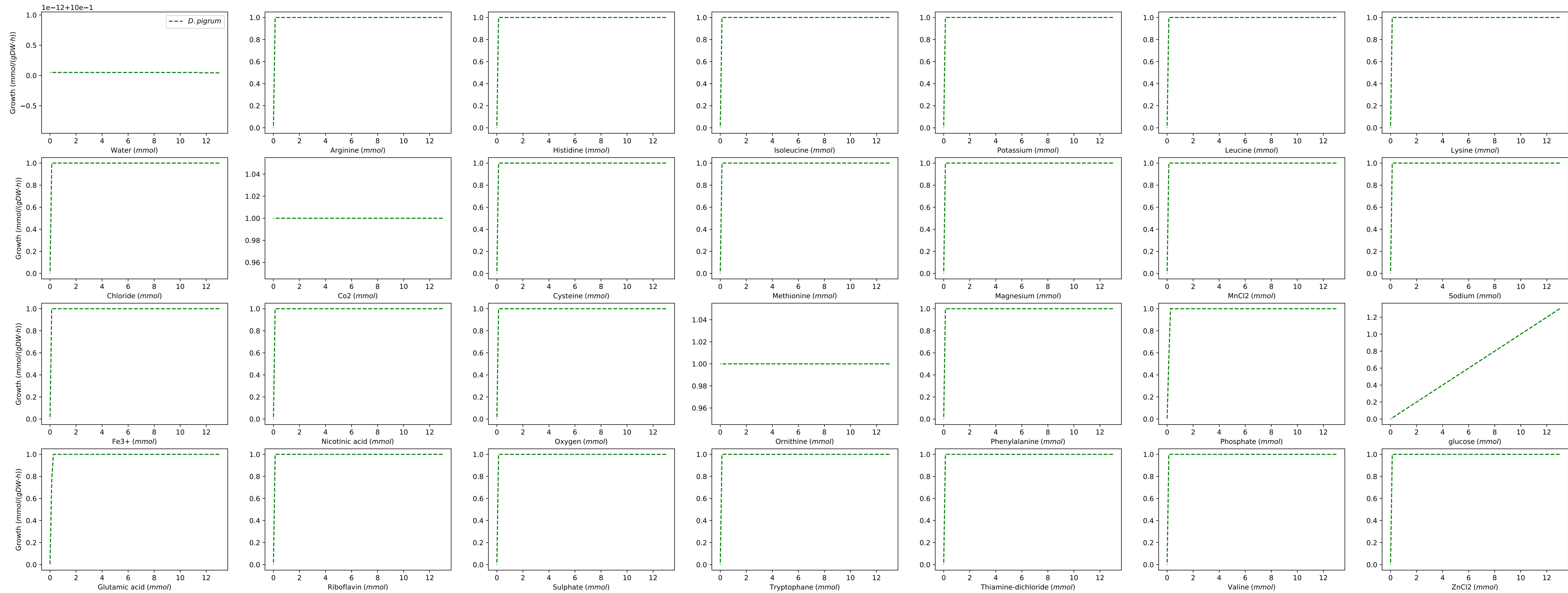

Supplement: Supplementary file 2 [file DataSheet_2.pdf]

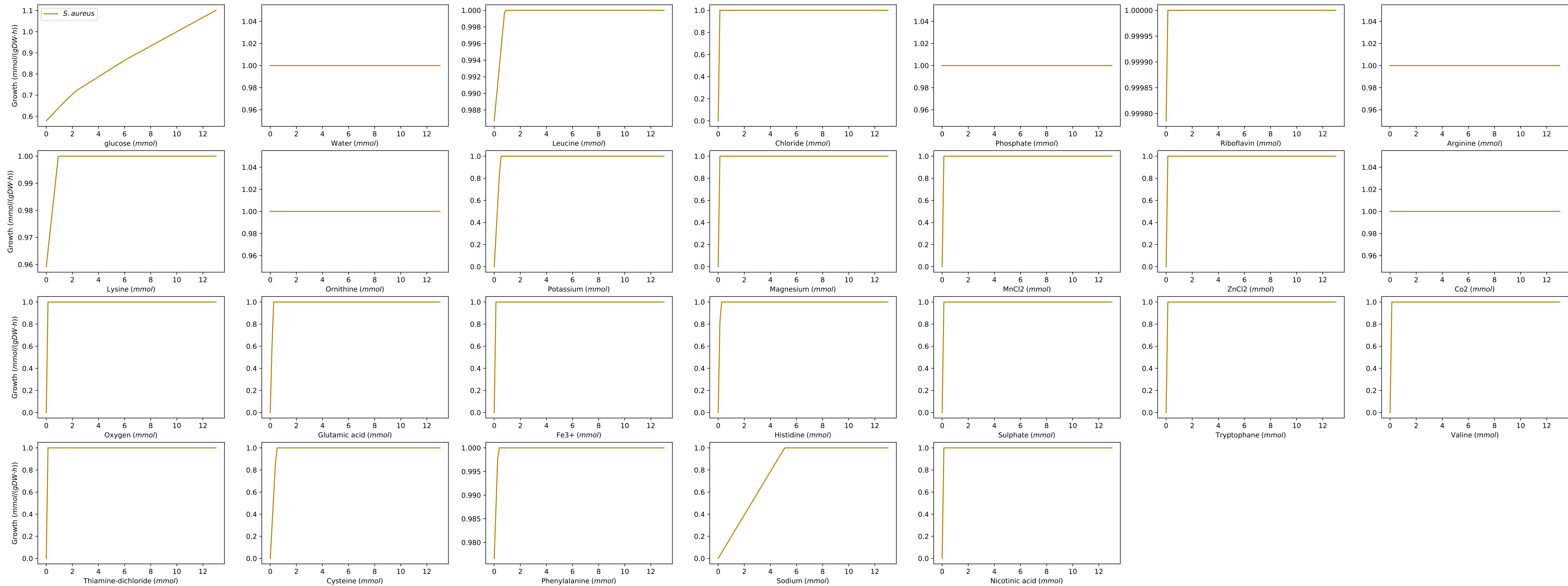

Supplement: Supplementary file 3 [file DataSheet_3.pdf]

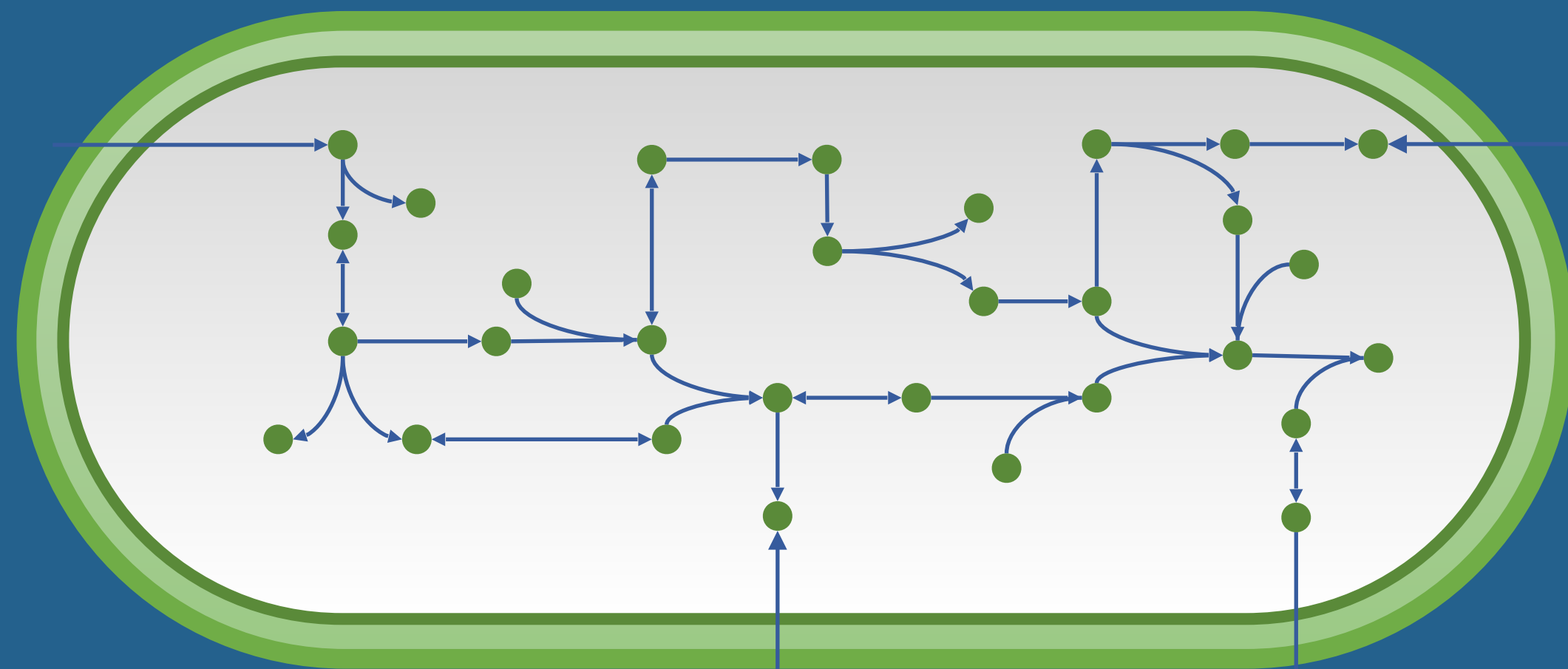

*D. pigrum*

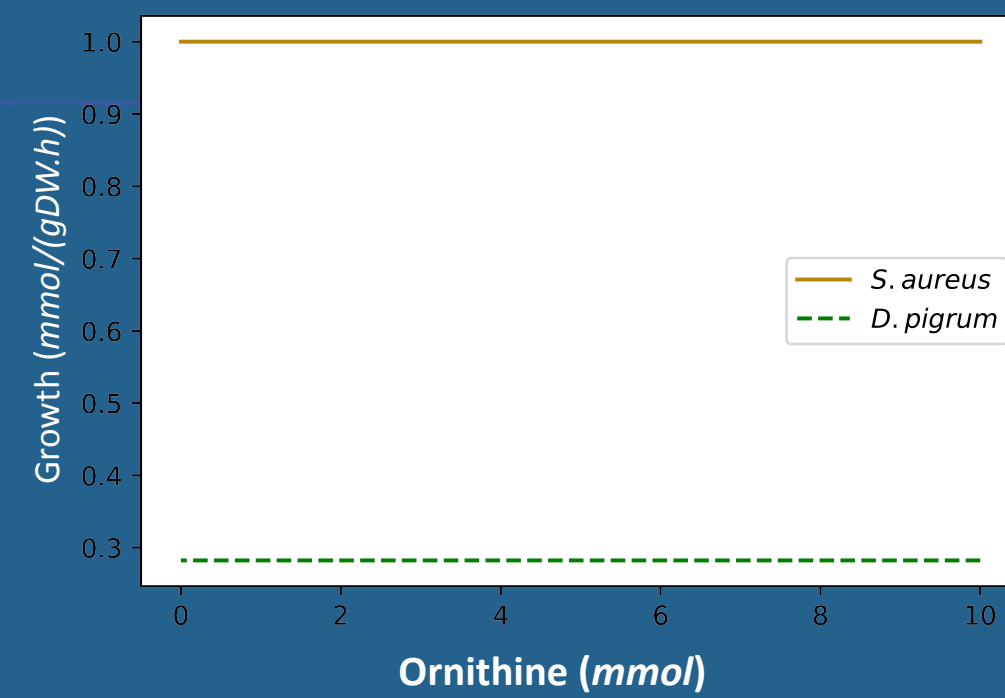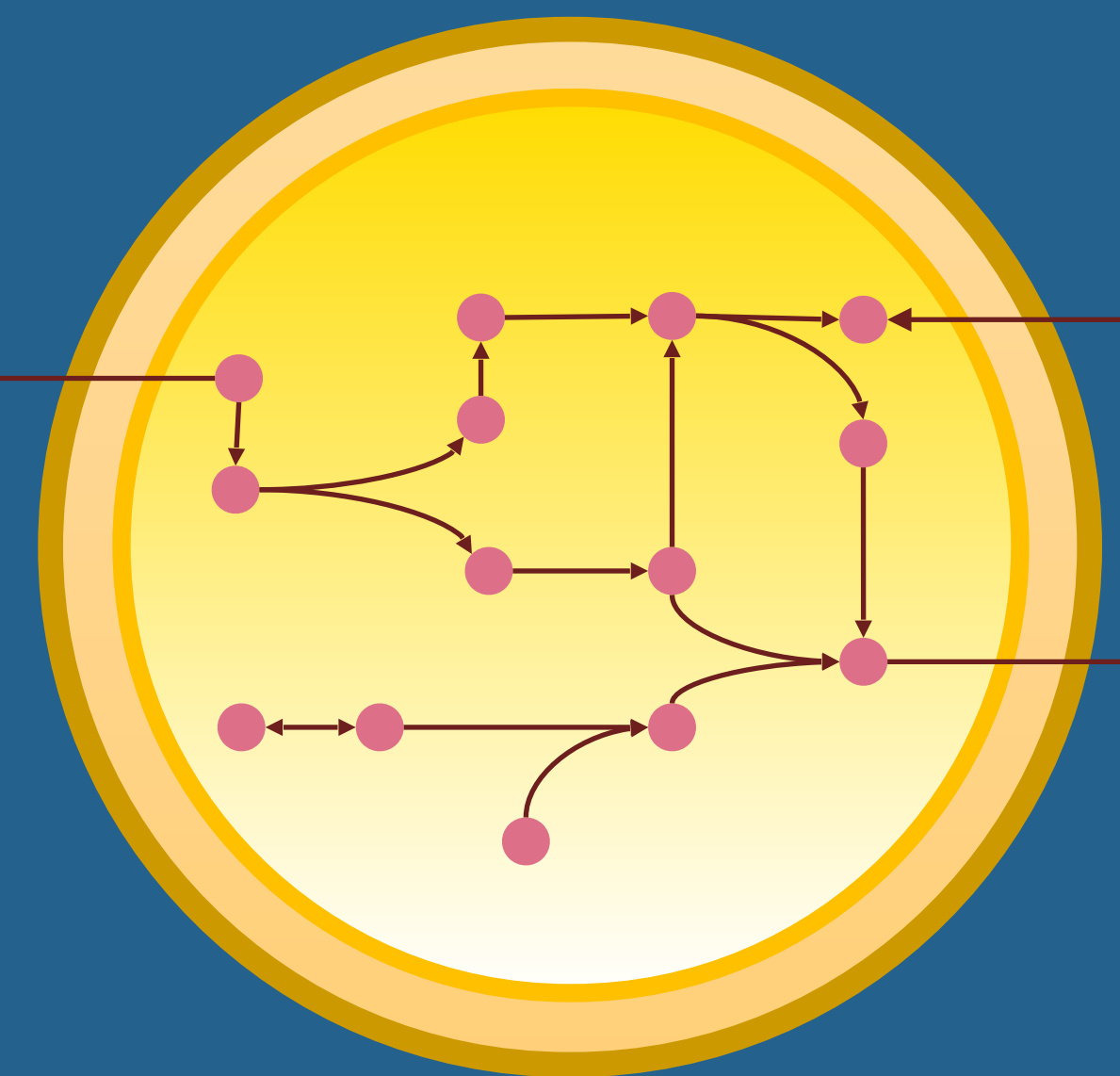

*S. aureus*

Supplement: Supplementary file 4 [file DataSheet_4.pdf]

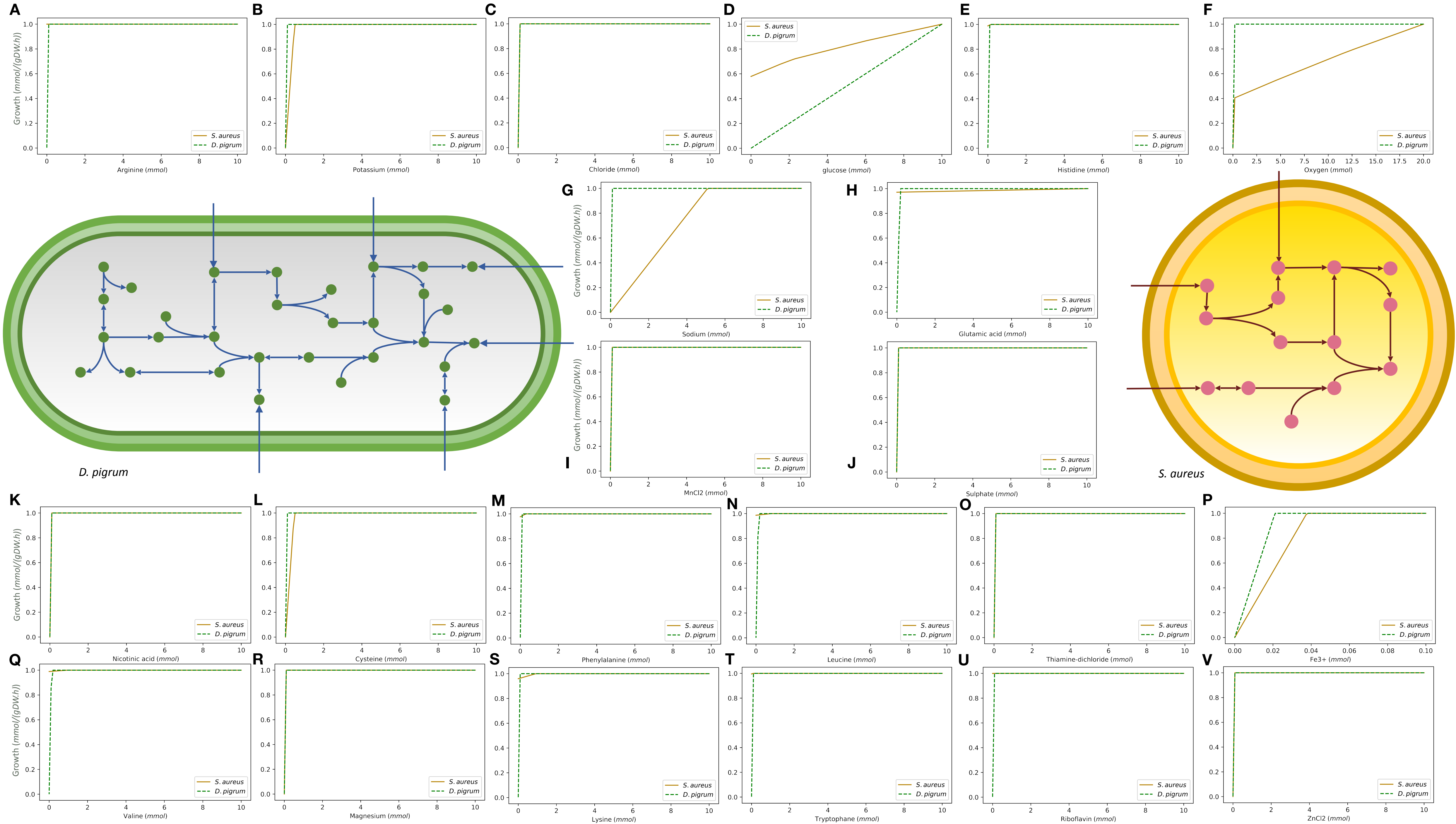

Supplement: Supplementary file 5 [file DataSheet_5.pdf]
